# Methylviologen resistance in loss-of-function mutants of the polyamine transporter gene *OsLAT5*

**DOI:** 10.1371/journal.pone.0346828

**Published:** 2026-04-16

**Authors:** Kyrylo Schenstnyi, Zhengzhi Zhang, Bo Liu, Masayoshi Nakamura, Van Schepler-Luu, Eliza P. I. Loo, Bing Yang, Wolf B. Frommer

**Affiliations:** 1 Heinrich Heine University Düsseldorf, Faculty of Mathematics and Natural Sciences, Institute for Molecular Physiology, Düsseldorf, Germany; 2 Division of Plant Science and Technology, Bond Life Sciences Center, University of Missouri, Columbia, Missouri, United States of America; 3 Institute for Transformative Bio-Molecules (WPI-ITbM), Nagoya University, Nagoya, Japan; 4 Present address: International Rice Research Institute, Pili Drive, Los Baños, Laguna, Philippines; 5 Donald Danforth Plant Science Center, St. Louis, Missouri, United States of America; South China Agricultural University, CHINA

## Abstract

TALENs and CRISPR/Cas have become routine tools for genome editing. During stable plant transformation, genes coding for editing enzymes, e.g., Cas9, guide RNAs (gRNA), and selectable or screenable markers are integrated into the nuclear genome. Identification of successful transformants relies on selectable or screenable markers, typically genes providing resistance to herbicides or antibiotics. Selectable markers use a substantial portion of the T-DNA, hence reducing transfer efficiency by limiting the effective number of TALENs or guide/pegRNAs that can be used. Marker genes are frequently subject to gene silencing. Here, we generated loss-of-function mutations in PUT/LAT-type polyamine transporter family genes to confer resistance to methylviologen (MV) in rice. As proof of concept, CRISPR/Cas9 constructs with gRNAs were generated to target three close homologs, namely *OsLAT1*, *OsLAT5*, and *OsLAT7*. Loss of *OsLAT5* (also known as *OsPUT3* or *OsPAR1*) function was sufficient to confer resistance to MV in rice seeds, seedlings and calli. Loss-of-function alleles generated by editing of *LAT5* can serve as a selectable marker at the seed germination stage. We discuss the potential utility of rice *lat5* loss of function variants as selectable markers for genome editing.

## Introduction

Genome editing is widely used in both model plant and crop species for research and biotechnology [1,2]. Combinations of CRISPR/Cas9/Cpf1 and prime editing have successfully generated elite rice varieties with broad-spectrum resistance to bacterial blight and other diseases [3–9]. Major advantages of genome editing over classical breeding include the precision of prime editing, speed, and the ability to target multiple traits simultaneously without linkage drag. At present, most genome editing approaches in plants require stable transformation with a CRISPR construct. Due to the low transformation efficiencies, screenable or selectable markers have to be implemented on the same construct [10,11]. A selectable marker typically comprises a cassette consisting of a strong ubiquitous plant promoter, an antibiotic or herbicide resistance gene, and a terminator. Commonly used selectable marker cassettes are comparatively large, using up a substantial fraction of the *Agrobacterium* T-DNA, and therefore, limiting the editing capacity for multiplex genome editing. Editing could be used to generate novel selectable markers, i.e., by introducing loss-of-function mutations of host genes, as an alternative.

The capacity/insert size of Agrobacterium T-DNA is 5–25 kb. The insertion of T-DNA into the host chromosome is initiated from the right border, and there is a length-dependent decrease in efficiency of the insertion of sequences towards the left border [12]. When the size of the components necessary for replication and selection in bacteria, and the size of *Cas9*/*Cpf1* modules are taken into account, the number of gRNAs that can be included into a single CRISPR construct, i.e., for multiplex genome editing, is limited by the size of a selectable marker for *in planta* selection. To fully utilize the T-DNA capacity available for gRNAs, we hypothesize that a selectable marker can be created by editing host plant’s endogenous trait genes. In the simplest case, loss of a gene function can lead to acquisition of a new trait, i.e., resistance to a chemical, metabolite or xenobiotic. The gain of a new trait can also serve as a control for successful editing. Such loss-of-function markers generated by editing could be beneficial also for transgene-free genome editing approaches [13–15]. To avoid potential issues associated with *Agrobacterium*-mediated T-DNA delivery, chemically synthesized gRNA and purified Cas proteins pre-assembled into ribonucleoproteins (RNP) can be used for transient transgene-free genome editing in plant cells [16,17]. Despite the availability of studies reporting successful transgene-free genome editing, transformation efficiencies remain too low to edit without selection [16–21].

In a search for potential selectable markers that are based on loss-of-function mutations introduced by editing, we chose rice homologs of the paraquat resistance genes originally identified in Arabidopsis as L-type amino acid transporters (LAT) [22–24]. Two *Arabidopsis* chloroplast-localized LAT proteins, namely AtLAT1 and AtLAT4 [25], had previously been reported to be involved in the transmembrane transport of polyamines (PA) and a phytotoxin N,N′-dimethyl-4,4′-bipyridinium dichloride methylviologen (MV; trademark paraquat; PQ) [22,23,26]. Alternative denominations for genes encoding LAT proteins include *RMV* (*Resistant to Methyl Viologen*), *PUT* (*Polyamine Uptake Transporter*), *LHR* (*Lower expression of Heat-responsive Gene*), and *PAR* (*Paraquat resistant*) [26].

Among the nine rice paralogs encoding LAT proteins [22–24], we focused on *LAT1/PUT1*, *LAT5/PUT3/PAR1*, and *LAT7/PUT2*, because triple rice *lat1/put1*, *lat5/put3/par1*, *lat7/put2* mutants were resistant to MV [27]. Furthermore, knockdown of *OsLAT5/OsPUT3/OsPAR1* via RNAi caused partial MV resistance resulting into reduction of chlorophyll content (≈ 20–35%) when plants were treated with the relatively high concentrations of MV (5 μM) [28]. Further studies also reported that among the afore mentioned three rice LAT proteins, only LAT5/PUT3/PAR1 localizes to chloroplast, similar to AtLAT1/AtPU3/AtRMV1 and AtLAT4/AtPU2/AtPAR1 [22,25,28,29]. Based on the prior RNAi evidence, subcellular localization, and functional role in MV transport, we hypothesized that *OsLAT5/OsPUT3/OsPAR1* could be a suitable selectable marker for genome editing approaches.

To test the hypothesis, we generated individual mutants in *OsLAT1/OsPUT1*, *OsLAT5/OsPUT3/OsPAR1*, and *OsLAT7/OsPUT2*, and found that a loss-of-function mutation in *OsLAT5/OsPUT3/OsPAR1* was sufficient to enable selection in axenic cultures during callus induction and regeneration of plants from calli as well as selection of T1 plants during germination. Notably, loss-of-function mutations in the two close paralogs *OsLAT1/OsPUT1* and *OsLAT7/OsPUT2* did not convey resistance under these conditions. The single mutants characterized here did not show apparent growth or developmental defects under our growth conditions. We therefore surmise that the generation of loss-of-function mutations by editing in the single *OsLAT5/OsPUT3/OsPAR1* locus is sufficient to obtain MV resistance, and we show that MV can be used as a selectable marker during callus induction and regeneration of plants from calli to identify transformants or in the seedling stage to detect lines carrying the edited locus.

## Materials and methods

### Sequence alignments and phylogenetic analysis

Protein sequences for the respective *Arabidopsis* and rice PUT/LAT-type transporters were extracted from UniProt and MSU: AtLAT1/AtPUT3/AtRMV1 (Q9FFL1), AtLAT3/AtPUT1 (Q9C6S4), AtLAT4/AtPUT2/AtPAR1 (Q9C6S5), AtPUT4 (Q9LHN7), AtPUT5 (Q9LH39), OsLAT1/OsPUT1 (Q6Z8D0); OsLAT2 (Q7Y166), OsLAT3 (Q10KQ0), OsLAT4 (A2XHA5), OsLAT5/OsPUT3/OsPAR1 (Q10HT5), OsLAT6 (A0A0P0XI35), OsLAT7/OsPUT2 (A0A0N7KU97), OsLAT8 (MSU: LOC_Os01g19850), and OsLAT9 (Q6Z0E2). Alignment of multiple amino acid sequences were generated with Clustal Omega [30]. The multiple sequence alignment output was processed by the pyBoxshade program to visualize the conserved amino acids within the analyzed sequences of rice and Arabidopsis PUT/LAT-type transporters. Non-conserved N- and C-termini (positions 1–138 and 744–1110) were trimmed from the multiple sequence alignment before they are used for the phylogenetic analysis. The phylogenetic tree was built using PHyML plug-in for Geneious Prime 2023.1.1 software using maximum-likelihood principle and 1000 bootstrap replications (*B* = 1000) [31]. The distantly related plasma membrane-localized amino acid permease protein AtAAP1 (Q84MA5) was used as an outgroup [32].

### Rice cultivation

Rice seed germination and plant cultivation were done according to the published protocol [33]. Briefly, rice seeds were sterilized and germinated on ½-salt strength MS medium (2.2 grams / liter of Murashige and Skoog basal salt mixture including vitamins from Duchefa Biochemie; Catalog # M0222) supplemented with 1% sucrose (Duchefa Biochemie; Product # S0809). Seedlings were grown for 10 days in tissue culture vessels (GA-7 Vessel, Magenta LLC) before transfer to soil. Plants were grown in greenhouses maintained at 30°C day / 25°C night, relative humidity (RH) 50−70% with supplemental LED lights (Valoya, BX100 NS1) following the 8h light / 16h dark light cycle (400 μmol/m^-2^s^-1^). Plants were fertilized weekly from the 2^nd^ week and biweekly from the 6^th^ week after germination with Peters Excel CalMag Grower 15:5:15 + 7CaO + 3MgO + TE fertilizer (ICL Specialty Fertilizers).

### CRISPR constructs

Four vectors were used to generate three CRISPR-Cas9 constructs targeting *OsLAT1*, *OsLAT5*, and *OsLAT7*, respectively. Modular intermediate vectors ptGgRNA1 and ptGRNA-T2 were used to make two guides. The recipient vector pENTR4-U6.1P-ccdBchl was used to assemble the gRNA units. Subsequently the assembly was mobilized into the binary Gateway vector pBY02rCas9-GW [34]. To target *OsLAT5*, one pair of complementary oligos with different four-base overhangs at the 5´-ends (gLAT5-F1 and gLAT5-R1) were denatured and annealed to form a double-stranded DNA fragment (dsOligo), which was cloned into ptGRNA1 at *BsmB*I sites. Similarly, a pair of oligonucleotides (gLAT5-F2 and gLAT5-R2) were cloned into ptGRNA-T2. After confirmation of successful insertion of the dsOligos, the two resulting plasmids were used along with pENTR4-U6.1P-ccdBchl to perform Golden Gate reactions using *Bsa*I and T4 ligase, resulting in substitution of *ccdBchl* cassette by the two gRNA units (tRNA-gRNA architecture) under control of rice *U6* promoter (U6.1P). The tRNA-gRNA cassette, flanked by *att*L1-*att*L2, was integrated into pBY02rCas9-GW via LR reactions (Invitrogen Gateway™ LR Clonase™ II Enzyme mix; Catalog # 11791020). The final plasmid (pBY02rCas9_gLAT5) was restricted with *BamH*I, *Hind*III to validate the correct insert size. Using the same approach but with different oligo pairs, CRISPR constructs pBY02rCas9_gLAT1 and pBY02rCas9_gLAT7 targeting *OsLAT1* and *OsLAT7*, respectively, were generated. Sequences of oligonucleotides are provided Supporting Information Table S1 in S1 File. The sequence of the binary vector used for editing of the *OsLAT* genes are provided in S4 File.

### Stable rice transformation

The rice cultivar Kitaake (*Oryza sativa* subsp. *japonica*) was used for *Agrobacterium*-mediated transformation following the previously published method [35]. Briefly, *Agrobacterium* strain EHA105 carrying a CRISPR construct was used to infect embryo-derived rice calli for 3 days. The calli were selected on callus-inducing medium supplemented with Hygromycin B (50 mg/L; Sigma Aldrich) for two rounds (14 days each round). Hygromycin-resistant calli were cultured on regeneration medium for 1 or 2 rounds (2 weeks per round) for shoot initiation. Shoots were moved to rooting medium for root initiation and elongation before transferring to soil and were grown in the greenhouse at 28°C /12h light and 24°C / 12h dark photoperiod.

### Genotyping of edited rice lines

To simplify the screening of putative edited T0 plants and to avoid expenses on Sanger sequencing of amplicons from each T0 plant, the gRNA target sites / Cas9 cleavage sites were designed to overlap with cleavage sites of restriction enzymes following the previously published screening strategy [7]. Therefore, introduction of mutations by Cas9^sgRNA^ results in loss of restriction sites. To detect mutations, T0 and successive generation plants were sampled for DNA extraction using CTAB method [36]. Gene-specific primers were used for amplification of relevant genomic fragments via polymerase chain reaction (PCR). PCR-derived amplicons were restricted to identify the amplicons that lost restriction sites. Sanger sequencing of PCR products was used to determine mutations. Plantlets carrying mutations in target genes were grown to maturity for seed multiplication and further selected for homozygosity of targeted loci. Sequences of oligonucleotides used for genotyping are provided in Supporting Information Table S1 in S1 File.

### MV selection methods for seedlings and calli

Seeds were surface-sterilized with 70% ethanol for one minute, washed in 6.5% sodium hypochlorite (NaOCl) solution for 10 minutes, and washed three times with autoclaved Milli-Q water. For evaluation of MV sensitivity during seed germination, seeds were sown on ½-salt strength MS medium (2.2 grams / liter of Murashige and Skoog basal salt mixture including vitamins from Duchefa Biochemie; Catalog # M0222) supplemented with 1% sucrose (Duchefa Biochemie; Catalog # S0809), and either containing autoclaved Milli-Q water as a solvent, or methyl viologen dichloride hydrate (C_12_H_14_Cl_2_N_2_ x H_2_O, Sigma-Aldrich; Catalog # 856177-1G). 10 mM stock solution of MV was stored at −20°C. Nine days after sowing on ½-salt strength MS medium, seedling shoot length was measured with a metric ruler (*n* = 16). For evaluation of MV sensitivity during seedling transfer, seeds were sown on ½-salt strength MS medium supplemented with 1% sucrose. Four days after seed sowing, seedlings were transferred to ½-salt strength MS medium supplemented with 1% sucrose, either containing solvent (autoclaved Milli-Q water), or methyl viologen dichloride hydrate. Five days after the transfer, seedling shoot length was measured with a metric ruler (*n* = 16). For evaluation of MV sensitivity during callus induction, seeds were sown on callus-inducing medium; calli started to emerge after about 10 days of cultivation (*n* > 16). Calli were transferred to the fresh callus-inducing medium three times in intervals of two weeks. The recipe for callus-inducing media was published previously [35].

### Statistical analyses

If not stated otherwise, graphical representations and statistical analyses were prepared using R 4.3.0 on RStudio 2021.09.2. Data were drawn as boxplots using package ggplot2 (https://ggplot2.tidyverse.org). The boxplot is delimited by the first and the third quartile of the distribution of the studied variable. The line inside the boxplot represents the median of the variable. Finally, the two lines that start from the boxplot join the minimum and maximum theoretical values. Each recorded datapoint is represented by the black or red dot. Red dots show the recorded datapoint for a seedling that was used for a photo. The total number (*n*) of biological samples for each genotype and condition equals 16. Statistics were calculated using Pairwise Wilcoxon Rank Sum Test with the p-value correction for multiple testing using Bonferroni adjustment method.

## Results

### Independent editing of three *AtLAT1* paralogs in rice

The rice genome contains nine PUT/LAT-type transporter-encoding genes [22–24]. A previous study had demonstrated that the triple knockout of rice *LAT1/PUT1, LAT5/PUT3/PAR1*, and *LAT7/PUT2* confers tolerance to MV [27], however it was unknown whether knockout of individual rice PUT/LAT-type transporter-encoding genes leads to the same phenotype. We hypothesized that loss-of-function mutations in one of these three closely related rice *LAT* genes would result into MV resistance and could be used as a selectable marker for genome editing approaches in rice.

**Fig 1:**
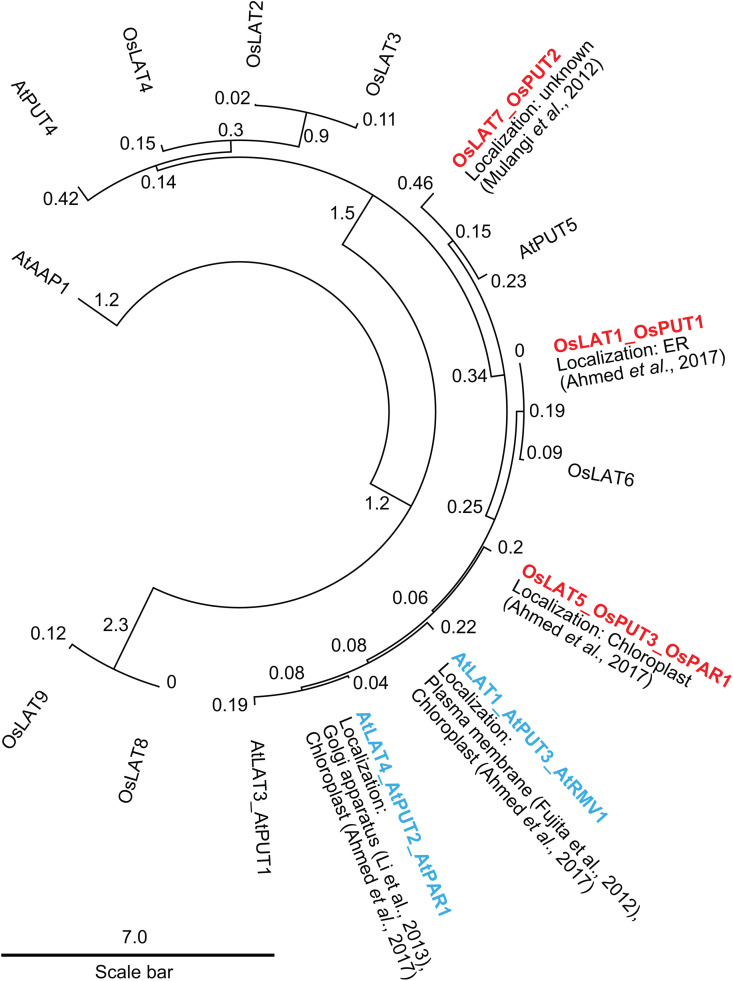
Rice LAT5 is most closely related to AtLAT1/AtPU3/AtRMV1 and AtLAT4/AtPU2/AtPAR1. Maximum-likelihood phylogenetic analysis of LAT proteins from rice and *Arabidopsis thaliana*. Arabidopsis LAT proteins that are known to confer MV resistance are colored in **blue** [23,28]. Rice LAT proteins that were selected for this study are highlighted in **red**. Values on tree branches represent substitutions per site based on 1000 bootstrap replications (*B* = 1000). Plasma membrane-localized amino acid permease protein AtAAP1 is used as an outgroup [32].

Based on a phylogenetic analysis of rice and Arabidopsis LAT proteins, OsLAT5/OsPUT3/OsPAR1 is the closest homolog of AtLAT1/AtPU3/AtRMV1 and AtLAT4/AtPU2/AtPAR1 (Fig 1 and S2 File); and among the analyzed rice LAT proteins, only OsLAT5/OsPUT3/OsPAR1 localizes to the plastids, similar to AtLAT1/AtPU3/AtRMV1 and AtLAT4/AtPU2/AtPAR1 [22,25,28,29]. Moreover, only *OsLAT5/OsPUT3/OsPAR1* was found to be expressed in multiple rice tissues, including leaves, roots, and callus (Supporting Information S3A in S3 File) [37], making it a potential candidate for the use as a selectable marker for genome editing approaches in rice. Specifically, an RNA-seq experiment reported FPKM (Fragments Per Kilobase of transcript per Million mapped reads) values of 25 in both leaves and roots and >100 in calli for *LAT5*/*PUT3*/*PAR1*. By comparison, *LAT1*/*PUT1* had substantially lower mRNA levels in leaves roots and calli (Supporting Information S3B in S3 File). *LAT7*/*PUT2* was also low in leaves and roots, but had FPKM values of about 24 in calli (Supporting Information S3C in S3 File).

**Fig 2:**
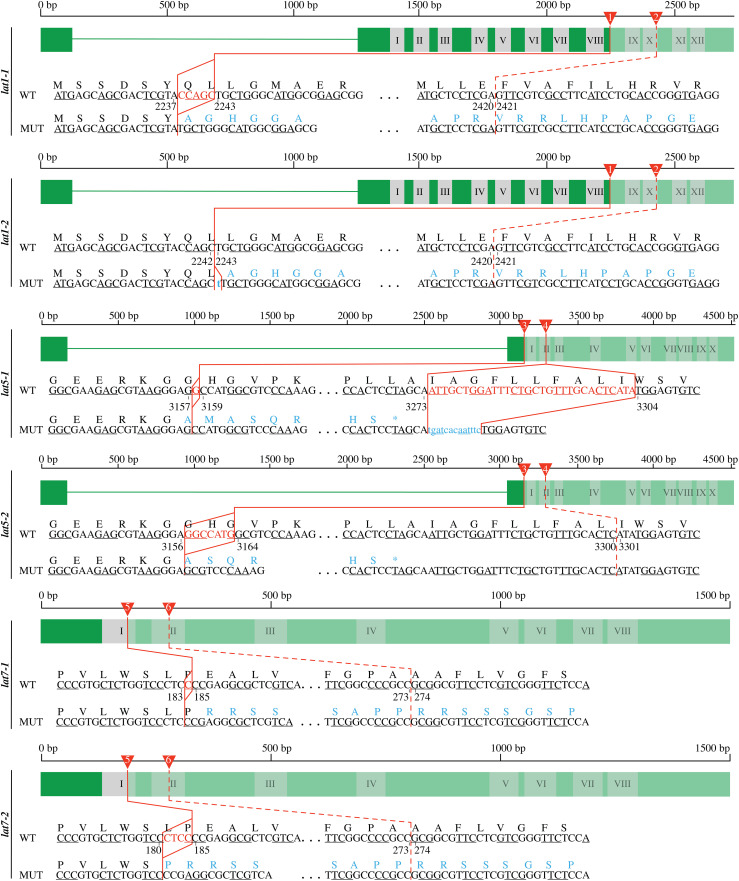
CRISPR/Cas9-mediated mutagenesis yields null *LAT1*, *LAT5*, and *LAT7* alleles. Graphical display of the CRISPR/Cas9-induced null *LAT1*, *LAT5*, and *LAT7* alleles. Black lines over green blocks provide information about the length of genomic sequences of each WT allele in base pairs (bp). Wide green horizontal bars indicate exons while green horizontal lines indicate introns. Grey shaded blocks with Roman numbers represent transmembrane domains according to the annotations from UniProt database. Faded colors indicate regions of a given mutant allele that are translated differently than a corresponding WT allele. Numbered red triangles indicate sequence parts targeted by gRNAs that were used to introduce mutations. Straight red lines highlight sequence parts that were mutated. While dash red lines represent sequence parts that were targeted by a gRNA, but were not mutated. The red font indicates deletions (upper case letters) and while insertions are highlighted with blue font (lower case letters). Blue indicates amino acids or translational stop codons (asterisk) that are changed due to frameshifts. Details on complete nucleotide and amino acid sequences of mutant alleles are provided in S5 and S6 File.

To evaluate whether loss-of-function mutations in one of three closely related *LAT* genes could be used for selection of transformants during rice transformation, *O. sativa* cv. Kitaake was transformed using *Agrobacterium*-mediated method with T-DNA constructs containing two gRNAs targeting either *LAT1/PUT1, LAT5/PUT3/PAR1* or *LAT7/PUT2*, respectively. The gRNAs target sequences were designed to lead to disruption of transmembrane spanning domains (Fig 2 and S5 File). Target sites in the transmembrane domains were chosen instead of the start codon, since disruption of transmembrane domains typically leads to loss of function, and editing of the start codon, in certain cases, will not cause loss of function due to the presence of alternative downstream translation start sites [38]. Two independent mutant alleles per each target gene were used for further experimentation (Fig 2 and Table 1). Mutations at target genes and putative protein sequences of the edited sequences are listed in Supporting Information S5 and S6 Files.

**Table 1:** Summary of loss-of-function mutations in *LAT1*, *LAT5*, and *LAT7* genes used in this study.

Allele	Type of mutations	Generation used for MV screening
*lat1−1*	Single site mutation: 5 bp deletion; frameshift	T2
*lat1–2*	Single site mutation: 1 bp insertion; frameshift	T3
*lat5−1*	Mutations at two sites: 1 bp deletion and 30 bp indel; frameshift	T4
*lat5−2*	Single site mutation: 7 bp deletion; frameshift	T2
*lat7−1*	Single site mutation: 1 bp deletion; frameshift	T3
*lat7−2*	Single site mutation: 4 bp deletion; frameshift	T3

### MV resistance of seedlings carrying *LAT5* null alleles

**Fig 3:**
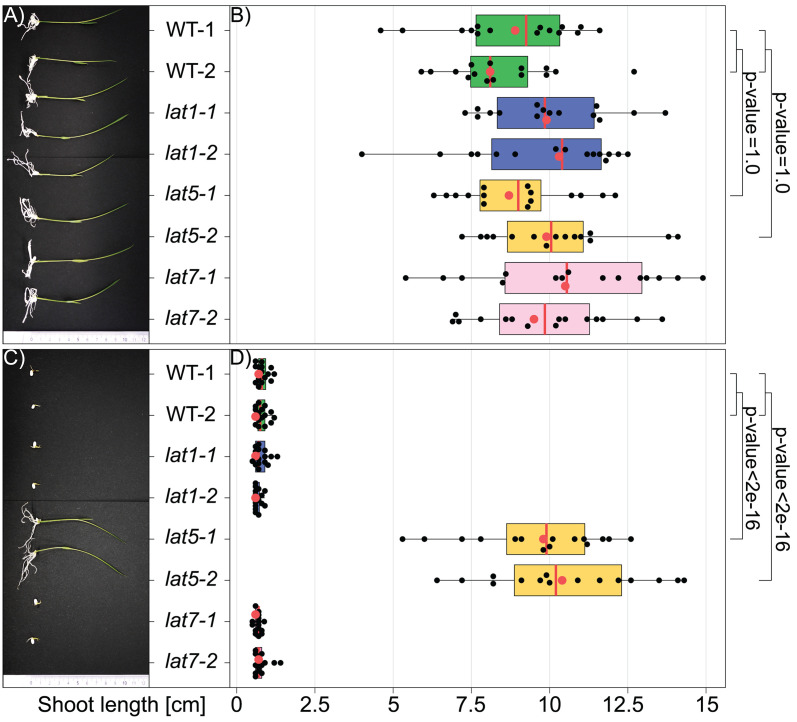
Loss-of-function mutations in *LAT5* lead to MV resistance. (A) WT, *lat1*, *lat5*, and *lat7* seedlings germinated on medium lacking MV (0 μM). (B) Quantification of MV resistance of WT, *lat1*, *lat5*, and *lat7* seedlings germinated on medium lacking MV (0 μM). (C) WT, *lat1*, *lat5*, and *lat7* seedlings germinated on medium containing MV (0.05 μM). (D) Quantification of MV resistance of WT, *lat1*, *lat5*, and *lat7* seedlings germinated on medium containing MV (0.05 μM). WT-1 and WT-2 are two independent lines of *O. sativa* cv. Kitaake harvested in 2022 and 2023, respectively. Each mutant genotype, i.e., *lat1*, *lat5*, and *lat7*, is represented by two independent lines homozygous for respective mutations (Fig 2 and Table 1). *n* = 16, where *n* is a number of seedlings representing each genotype germinated on each medium. All dots represent values for “Shoot length, cm” for individual plantlets, while red dots represent these values for plantlets that were chosen for pictures in (A) and (C). Box plots: red vertical line is median; box limits are lower and upper quartiles; whiskers are highest and lowest data points. Pairwise Wilcoxon Rank Sum Test with the p-value correction for multiple testing using Bonferroni adjustment method was used to calculate significant differences between groups.

To evaluate the effect of the frameshift mutations in the three *LAT* genes on MV resistance, seeds of homozygous mutant lines were germinated on ½-salt strength MS medium supplemented either with the solvent (0 μM MV) or 0.05 μM MV. WT, *lat1*, and *lat7* seeds were equally sensitive to MV and no statistically significant differences in sensitivity to MV between these genotypes were detected (Fig 3). Only *lat5−1* and *lat5−2* seeds germinated on MV-containing medium indicating that a loss-of-function mutation in *LAT5* is sufficient to provide resistance to MV.

**Fig 4:**
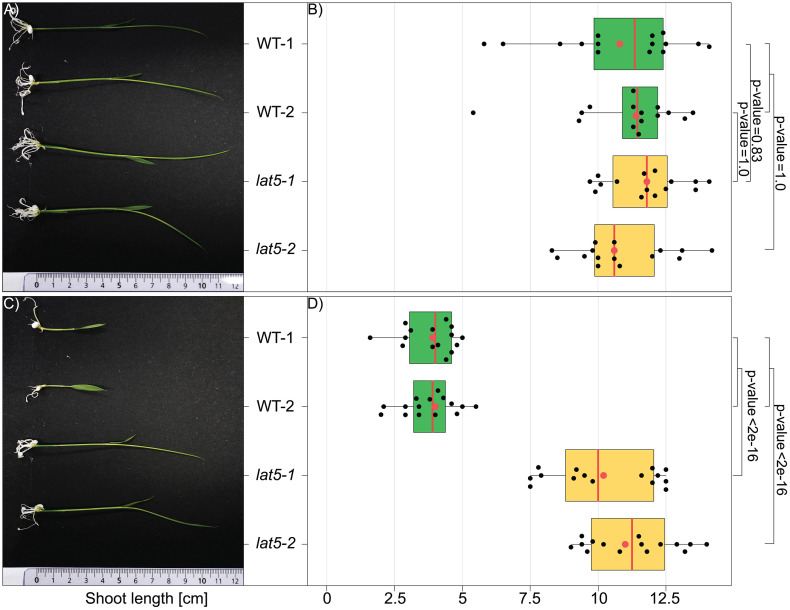
Shoots carrying null *LAT5* alleles regenerate on MV-containing medium. (A) WT and *lat5* seedlings regenerated on medium lacking MV (0 μM). (B) Quantification of seedling length of WT and *lat5* seedlings regenerated on medium lacking MV (0 μM). (C) WT and *lat5* seedlings regenerated on medium containing MV (0.1 μM). (D) Quantification of seedling length of WT and *lat5* seedlings regenerated on medium containing MV (0.1 μM). WT-1 and WT-2 are two independent lines of *O. sativa* cv. Kitaake harvested in 2022 and 2023, respectively. *lat5−1* and *lat5−2* are two independent lines homozygous for respective loss-of-function mutations in *LAT5* (Fig 2 and Table 1). Shoot length (cm) for 16 seedlings per genotype / treatment was quantified (*n* = 16). All dots represent values for “shoot length, cm” for individual plantlets, while red dots represent these values for plantlets that were chosen for pictures in (A) and (C). Box plots: red vertical line is median; box limits are lower and upper quartiles; whiskers are highest and lowest data points. Pairwise Wilcoxon Rank Sum Test with the p-value correction for multiple testing using Bonferroni adjustment method was used to calculate significant differences between groups.

Since a loss-of-function mutation in *LAT5* was sufficient to provide MV resistance, MV-based selection of primary transformants carrying null *LAT5* alleles after transgenic and transgene-free genome editing might be applicable also during shoot regeneration process. To test this hypothesis, WT and *lat5* seeds were sown on ½-salt strength MS medium. After four days, WT and *lat5* seedlings were transferred onto ½-salt strength MS medium supplemented either with the solvent (0 μM MV) or 0.1 μM MV. Five days after transfer only lines that contained null *LAT5* alleles, namely *lat5−1* and *lat5−2*, continued development on MV-containing medium, while growth of WT seedlings was inhibited (Fig 4). These data indicate that loss-of-function mutations in *LAT5* can be used for selection of primary transformants/edited lines during shoot regeneration stage either after transgenic or transgene-free genome editing.

### Selection of MV resistance in rice *lat5* calli

**Fig 5:**
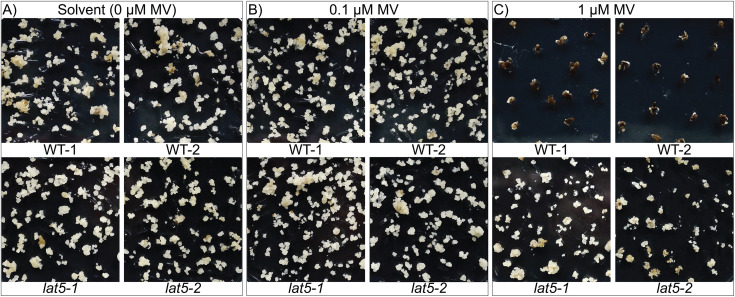
MV does not inhibit callus formation on *lat5* mature embryos. WT and *lat5* mature embryos were germinated on callus-inducing medium supplemented with either (A) the solvent (0 μM MV), (B) 0.1 μM MV or (C) 1 μM MV. WT-1 and WT-2 are two independent lines of *O. sativa* cv. Kitaake harvested in 2022 and 2023, respectively. *lat5−1* and *lat5−2* are two independent lines homozygous for respective loss-of-function mutations in *LAT5* (Fig 2 and Table 1).

Even though we have demonstrated that loss-of-function mutations in *LAT5* can be used for selection of photosynthetic young seedlings and germinating seeds (Figs 3C and 4C), transgenic and non-transgenic genome editing approaches in rice rely on selection of non-photosynthetic calli during callus induction and propagation, i.e., stages preceding shoot regeneration [14,33]. Previously, MV had been shown to also be effective on non-photosynthetic dark-grown kidney bean cells [39]. To test whether the MV selection can be used during callus transformation to identify primary transformants, WT and *lat5* seeds were sown on callus-inducing medium supplemented either with solvent (0 μM MV), 0.1 μM MV or 1 μM MV. WT and *lat5* seeds germinated normally on all media. Six weeks after seeding *lat5* calli were formed on all tested media, while WT calli developed only on the callus-inducing media containing solvent and 0.1 μM MV (Fig 5). No WT calli were formed on the medium containing 1 μM MV. These data indicate that MV inhibits growth of photosynthetically inactive rice tissues, such as WT callus.

Taken together, our results demonstrate that a loss-of-function mutation in *OsLAT5* is sufficient for providing resistance to MV in calli and seedlings, therefore, supporting a potential use of null *OsLAT5* alleles as selectable markers during callus and shoot regeneration processes.

## Discussion

Transgene-based editing with CRISPR/Cas9 and CRISPR/Cas12a is presently a popular technique for introducing targeted mutations in model species and crops [9]. Identification of transformant events relies on the introduction of selectable markers, typically genes encoding proteins that mediate resistance to antibiotics or herbicides [7,9,40,41]. A selectable marker that can be obtained by editing, i.e., based on loss of function of an endogenous gene, would be advantageous for transgene-dependent and, especially, for transgene-free genome editing approaches suffering from low transformation efficiencies [16,19,20,42,43].

Selectable markers that are based on editing of endogenous rice genes, namely OsALS^P171L^, OsALS^W548M^, OsALS^S627I^, and OsEPSPS1^IVS^ have recently been generated using base-editing or prime-editing approaches [40,44–46]. However, the afore mentioned selectable markers are based on edits within *OsALS* and *OsEPSPS1* coding sequences requiring specific amino acid substitutions, and therefore, these selectable markers might be generated with the low efficiency during editing with commonly used CRISPR/Cas9 and CRISPR/Cas12a endonucleases capable of introducing indels [47]. Other studies demonstrated that loss of *OsARF18* function causes a glufosinate resistance phenotype after leaf spraying [48]. Similarly, loss of *OsAFB4* function and loss of *OsHPPD* function leads to resistance to synthetic auxin picloram and mesotrione, respectively [49,50]. Here, we developed a recessive marker that is caused by loss of *OsLAT5*/*OsPUT3*/*OsPAR1* function, to confer resistance to the phytotoxin methylviologen (MV) as a method of selection *in vitro*.

A selection system based on the loss-of-function in *AtPAR1* had been previously established in Arabidopsis [15]. In parallel to our study, it was demonstrated that simultaneous disruption of three rice polyamine uptake transporter genes, namely *LAT1*/*PUT1*, *LAT5*/*PUT3*/*PAR1*, and *LAT7*/*PUT2*, confers resistance to MV [27]. Our data indicate that disruption of a single polyamine uptake transporter gene in rice, i.e., *LAT5*/*PUT3*/*PAR1*, is sufficient to provide resistance to MV (Figs 3C, 4C, and 5C). Our results are further supported by a study reporting that RNAi-based *LAT5/PUT3/PAR1* knockdown transgenic rice lines are resistant to MV upon spraying, while *LAT5/PUT3/PAR1*-overexpressing transgenic seedlings are hypersensitive to MV [28]. We hypothesize that loss-of-function mutations in *LAT1*/*PUT1* and *LAT7*/*PUT2* do not provide resistance to MV, due to the low base expression levels of these two genes, when compared to *LAT5*/*PUT3*/*PAR1* (S3 File) [37]; however alternative hypotheses, e.g., different substrate specificity, lower transport capacity or different subcellular localization, are also conceivable.

Because disruption of rice *LAT5*/*PUT3*/*PAR1* provides effective MV resistance (Figs 3C, 4C, and 5C), gRNAs that target regions that encode transmembrane helices might be used for selecting transformants in transgene-based and transgene-free genome editing experiments. We demonstrate MV-based selection in photosynthetically active rice tissues, e.g., germinating seeds (Fig 3C) and young seedlings (Fig 4C). MV resistance of green tissues is consistent with MV being known to transfer electrons from photosystem I to molecular oxygen, which leads to formation of cytotoxic reactive oxygen species (ROS) and photodestruction of chlorophyll [28,51]. Notably, MV-based selection was also successfully obtained for non-photosynthetically active rice calli (Fig 5). Callus was grown photoautotrophically in a medium supplemented with 3% of sucrose. The high sucrose levels in the media appeared to prevent cytotoxic symptoms on the WT callus cells grown on the medium containing 0.1 μM MV (Fig 5B) [52], while at 1 μM MV callus induction from WT mature embryos was inhibited effectively (Fig 5C). Our results are in line with the study which demonstrated that high MV concentrations inhibited growth of *Phaseolus vulgaris* cells during cultivation in darkness, likely by reducing DNA synthesis, and inhibiting the activity of enzymes involved in cellular defense against ROS [39]. The deleterious effects of MV on non-photosynthetic cells was attributed to iron [39].

Since 1 μM MV blocked callus induction from WT mature embryos (Fig 5C), MV-based selection of *lat5*/*put3*/*par1* calli directly after transformation or transfection might be possible. In the case of *Arabidopsis*, *PAR1* null alleles generated by CRISPR-Cas9 were shown to confer resistance to 1 µM and 10µM MV [15]. In our study reduction in MV concentrations enabled selection on 20–200-fold lower MV concentrations compared to the afore mentioned study [15], i.e., 0.05 µM and 0.1 µM in rice. At MV concentrations of 0.1 μM, a growth inhibition on neighboring calli cannot be excluded but appears to be minor or negligible.

Further evaluation of the use of loss-of-function mutations in *OsLAT5/OsPUT3/OsPAR1* as a selectable marker for base and prime editing approaches via introduction of a premature stop codon in *OsLAT5/OsPUT3/OsPAR1* during regeneration after transfection or transformation will be necessary. Since genome-editing, at least in rice, is highly efficient, frequently yielding biallelic mutations, e.g., high rates of multisite biallelic mutations were reported in several studies [33,34,53]. Therefore, a recessive selection marker, although likely to reduce the total number of events, will only be disadvantageous relative to dominant markers if editing efficacy is low, e.g., due to guide RNA design, or due to low transformation efficiencies, or due to monoallelic loss-of-function mutations in *OsLAT5/OsPUT3/OsPAR1*.

Identification of lines that do not carry a transgene is an essential prerequisite for the classification of edited lines to be treated equivalent to conventionally bred lines under suitable regulations [54]. However, one of the challenges is that *Agrobacterium*-mediated transformation can lead to partial insertion of T-DNA copies [12]. Another challenge is the inadvertent insertion of vector backbone fragments into the genome of transformed plants [55,56]. The evaluation of transgene removal is technically challenging since proving absence is impossible, due to technical detection limits and potential flaws that prevent detection [57]. For example, genotyping and whole genome sequencing of hornless bulls generated with the help of genome editing identified the unintended insertion of plasmid sequences into the genome [58–60]. Thus, without further analyses, it is not possible to conclude that hygromycin-sensitive MV-resistant Arabidopsis *par1* lines generated via *Agrobacterium*-mediated transformation are transgene-free [15]. Therefore, transgene-free (DNA-free) editing based on transfection with preassembled (Cas9^sgRNA^, Cas12a^cRNA^, base editor^sgRNA^, prime editor ^pegRNA/ngRNA^) RNP complexes or TALENs will be the new frontier to overcome the issues caused by *Agrobacterium*-mediated transformation.

One of the possible limitations for the use of rice *lat5/put3/par1* as a selectable marker via transgene-dependent or DNA-free editing approaches is the potential spread of MV resistance via horizontal gene transfer from the crop to weedy rice [61,62]. Therefore, elimination of *lat5/put3/par1* via backcrossing complemented with the MV resistance screens, DNA gel blotting, PCR-based screening, in each generation, followed by whole genome sequencing and additional tests stipulated by country-specific biosafety guidelines are the minimal suggested steps prior to commercialization of edited lines that are developed using *lat5/put3/par1* as a selectable marker [57,63].

## Conclusions

Altogether we demonstrate that generation of loss-of-function mutations by editing in the single *OsLAT5/OsPUT3/OsPAR1* locus is sufficient to obtain resistance to MV in rice calli, seedlings, and seeds. Notably, we suggest that this approach can be easily adapted to generate orthogonal selection in model species and crops. We conclude that loss-of-function mutations in *OsLAT5/OsPUT3/OsPAR1* locus may potentially be used as a selectable marker for transgenic and transgene-free RNP-mediated genome editing approaches during callus propagation and germination of seedlings on MV-containing media as well as spraying of MV on leaves of regenerated plantlets. We predict that loss-of-function mutations within *OsLAT5/OsPUT3/OsPAR1* locus might be generated not only by Cas9, but also by Cas12a, base editors, prime editors, TALEN, and other types of editors. Since it cannot be excluded that ROS generated in susceptible unedited *OsLAT5/OsPUT3/OsPAR1* cells will impact neighboring cells, next suggested steps are to evaluate the use of loss-of-function mutations in *OsLAT5 OsPUT3/OsPAR1* locus as a selectable marker directly during regeneration after transfection or transformation.

## Supporting information

S1 File Oligonucleotides used for assembly of CRISPR constructs and for genotyping of putative transformants. (DOCX)

S2 File Alignment of LAT/PUT protein sequences from Arabidopsis and rice. Alignments are made using Clustal Omega algorithm. Amino acids that are conserved in 60% of sequences are shaded in BLACK using pyBoxshade program. Non-conserved N- and C-termini (positions 1–138 and 744–1110) were trimmed from the multiple sequence alignment before they are used for the phylogeny analysis. Individual knockouts of *AtLAT1/AtPUT3/AtRMV1* (At5g05630) and *AtLAT4/AtPUT2/AtPAR1* (At1g31830) lead to MV resistance [23,28]. AtLAT1/AtPUT3/AtRMV1 and AtLAT4/AtPUT2/AtPAR1 are highlighted in **blue**. Knockdown of *OsLAT5/OsPUT3/OsPAR1* lead to MV resistance [28]. Triple mutants of *OsLAT1/OsPUT1*, *OsLAT5/OsPUT3/OsPAR1*, and *OsLAT7/OsPUT2* are resistant to MV [27]. OsLAT1/OsPUT1, OsLAT5/OsPUT3/OsPAR1, and OsLAT7/OsPUT2 are highlighted in **red**.
(DOCX)

S3 File Organ-specific mRNA levels for (A) *OsLAT5*/*OsPUT3*/*OsPAR1*, (B) *OsLAT1*/*OsPUT1* and (C) *OsLAT7*/*OsPUT2* [37]. FPKM: Fragments Per Kilobase of transcript per Million mapped reads.
(DOCX)

S4 File Vector maps used for knockout of individual OsLAT genes. The following parts of the T-DNA are annotated with colors: RIGHT AND LEFT BORDERS, *U6* PROMOTER, GLYCINE tRNA, gRNA TARGET SITES from the S1 Table, MAIZE *UBIQUITIN* PROMOTER, CAS9 coding sequence, NOPALINE SYNTHASE (NOS) TERMINATOR, CAULIFLOWER MOSAIC VIRUS 35S PROMOTER, *HYGROMYCIN PHOSPHOTRANSFERASE*.
(DOCX)

S5 File Alignments of coding sequences (CDS) of rice LAT1, LAT5, LAT7 wild-type and the mutant alleles used in this study. Alignments are made using MUSCLE (Multiple Sequence Comparison by Log- Expectation) algorithm. Non-mutated nucleotides that are shaded in BLACK using pyBoxshade program. Deleted nucleotides in mutant alleles are represented with the “–“ symbol. Inserted nucleotides in mutant alleles are non-shaded. Two guide RNAs were used for knock out of each rice gene. Selected guide RNAs are colored in **red**, while PAM sites are colored in **green**.
(DOCX)

S6 File Predicted OsLAT1, OsLAT5, and OsLAT7 protein sequences from single *oslat* mutant lines. (DOCX)

## Abbreviations

CRISPR/Cas9: Clustered regularly interspaced short palindromic repeats/CRISPR-associated protein-9 nuclease
GOI: Gene-of-interest
gRNA: Guide RNA
LAT: L-type Amino Acid Transporter
LHR: Lower expression of Heat-responsive Gene
MUSCLE: Multiple Sequence Comparison by Log- Expectation
MV: methylviologen
PA: Polyamines
PAR: Paraquat resistant
pegRNA: Prime editing guide RNA
PUT: Polyamine Uptake Transporter
ROS: Reactive oxygen species
RMV: Resistant to Methyl Viologen
RNP: Ribonucleoproteins.
