# Supplementary material for: Methylviologen resistance in loss-of-function mutants of the polyamine transporter gene OsLAT5
Source: PLoS One. 2026 Apr 16;21(4):e0346828. doi: 10.1371/journal.pone.0346828 (PMC13086316; doi:10.1371/journal.pone.0346828)
Supplement: S1 File — (DOCX) [file pone.0346828.s001.docx]

**Supporting Information S1. Oligonucleotides used for assembly of CRISPR constructs and for genotyping of putative transformants.**

| **#** | **Oligo name** | **Sequence 5’>3’** | **Purpose** |
| --- | --- | --- | --- |
| 1 | gLAT1-F1 | gcagCGACTCGTACCAGCTGCT | Assembly of CRISPR construct for *OsLAT1* knockout |
| 2 | gLAT1-R1 | aaacAGCAGCTGGTACGAGTCG |  |
| 3 | gLAT1-F2 | gcagGATGAAGGCGACGAACTCG |  |
| 4 | gLAT1-R2 | aaacCGAGTTCGTCGCCTTCATC |  |
| 5 | gLAT5-F1 | gcagGAAGAGCGTAAGGGAGGCCA | Assembly of CRISPR construct for *OsLAT5* knockout |
| 6 | gLAT5-R1 | aaacTGGCCTCCCTTACGCTCTTC |  |
| 7 | gLAT5-F2 | gcagTTCTGCTGTTTGCACTCATA |  |
| 8 | gLAT5-R2 | aaacTATGAGTGCAAACAGCAGAA |  |
| 9 | gLAT7-F1 | gcagTGCTCTGGTCCCTCCCCG | Assembly of CRISPR construct for *OsLAT7* knockout |
| 10 | gLAT7-R1 | aaacCGGGGAGGGACCAGAGCA |  |
| 11 | gLAT7-F2 | gcagACGAGGAACGCCGCGGC |  |
| 12 | gLAT7-R2 | aaacGCCGCGGCGTTCCTCGT |  |
| 13 | LAT1-F1 | CTACTGGGATTCGATCAGC | PCR-amplification of *OsLAT1* for genotyping CRISPR plants |
| 14 | LAT1-R1 | AGATCCGGGTTAACGGAGAA |  |
| 15 | LAT5-F1 | GTCAACTTAAAACTCGGATATG | PCR-amplification of *OsLAT5* for genotyping CRISPR plants |
| 16 | LAT5-R1 | ACCCAGCCAACTATTGTCAA |  |
| 17 | LAT7-F1 | CTCGTCGCGCTCATCTTCTA | PCR-amplification of *OsLAT7* for genotyping CRISPR plants |
| 18 | LAT7-R1 | CTCGCCTTGTCCCAGTAGTT |  |
