# Supplementary material for: Methylviologen resistance in loss-of-function mutants of the polyamine transporter gene OsLAT5
Source: PLoS One. 2026 Apr 16;21(4):e0346828. doi: 10.1371/journal.pone.0346828 (PMC13086316; doi:10.1371/journal.pone.0346828)
Supplement: S2 File — Alignments are made using Clustal Omega algorithm. Amino acids that are conserved in 60% of sequences are shaded in BLACK using pyBoxshade program. Non-conserved N- and C-termini (positions 1–138 and 744–1110) were trimmed from the multiple sequence alignment before they are used for the phylogeny analysis. Individual knockouts of AtLAT1/AtPUT3/AtRMV1 (At5g05630) and AtLAT4/AtPUT2/AtPAR1 (At1g31830) lead to MV resistance [23,28]. AtLAT1/AtPUT3/AtRMV1 and AtLAT4/AtPUT2/AtPAR1 are highlighted in blue. Knockdown of OsLAT5/OsPUT3/OsPAR1 lead to MV resistance [28]. Triple mutants of OsLAT1/OsPUT1, OsLAT5/OsPUT3/OsPAR1, and OsLAT7/OsPUT2 are resistant to MV [27]. OsLAT1/OsPUT1, OsLAT5/OsPUT3/OsPAR1, and OsLAT7/OsPUT2 are highlighted in red. (DOCX) [file pone.0346828.s002.docx]

**Supporting Information S2. Alignment of LAT/PUT protein sequences from Arabidopsis and rice.** Alignments are made using Clustal Omega algorithm. Amino acids that are conserved in 60% of sequences are shaded in BLACK using pyBoxshade program. Non-conserved N- and C-termini (positions 1-138 and 744-1110) were trimmed from the multiple sequence alignment before they are used for the phylogeny analysis. Individual knockouts of *AtLAT1/AtPUT3/AtRMV1* (At5g05630) and *AtLAT4/AtPUT2/AtPAR1* (At1g31830) lead to MV resistance [23, 28]. AtLAT1/AtPUT3/AtRMV1 and AtLAT4/AtPUT2/AtPAR1 are highlighted in **blue**. Knockdown of *OsLAT5/OsPUT3/OsPAR1* lead to MV resistance [28]. Triple mutants of *OsLAT1/OsPUT1*, *OsLAT5/OsPUT3/OsPAR1*, and *OsLAT7/OsPUT2* are resistant to MV [27]. OsLAT1/OsPUT1, OsLAT5/OsPUT3/OsPAR1, and OsLAT7/OsPUT2 are highlighted in **red**.

Sequence position 1 ........10........20........30........40........50........60
AtAAP1 1 ~~~~~~~~~~~~~~~~~~~~~~~~~~~~~~~~~~~~~~~~MASG................
**AtLAT1_AtPUT3_AtRMV1** 1 ~~~~~~~~~~~~~~~~~~~~~~~~~~~~~~~~~~~~~MTELSSP................
AtLAT3_AtPUT1 1 ~~~~~~~~~~~~~~~~~~~~~~~~~~~~~~~~~~~~~~~~~~~~~~~~~~~~~~~~~~~~
**AtLAT4_AtPUT2_AtPAR1** 1 ~~~~~~~~~~~~~~~~~~~~~~~~~~~~~~~~~~~~~~~~~~~~~~~~~~~~~~~~~~~~
AtPUT4 1 ~~~~~~~~~~~~~~~~~~~~~~~~~~~~~~~~~~~~~~~~~~~~~~~~~~~~~~~~~~~~
AtPUT5 1 ~~~~~~~~~~~~~~~~~~~~~~~~~~~~~~~~~~~~~~~~~~~~~~~~~~~~~~~~~~~~
**OsLAT1_OsPUT1** 1 MADTGGRPEV......SLATVRSPGHPAASTTAA..AAADLGHA................
OsLAT2 1 ~~~~~~~~~~~~~~~~~~~~~~~~~~~~~~~~~~~~~~~~~~~~~~~~~~~~~~~~~~~~
OsLAT3 1 ~~~~~~~~~~~~~~~~~~~~~~~~~~~~~~~~~~~~~~~~MSEL................
OsLAT4 1 ~~~~~~~~~~~~~~~~~~~~~~~~~~~~~~~~~~~~~~~~~~~~~~~~~~~~~~~~~~~~
**OsLAT5_OsPUT3_OsPAR1** 1 MTNAWISPSVVALCPSPLPSSRLPGSVLSCWPDSRGIRRGAGEG................
OsLAT6 1 ~~~~~~~~~~~~~~~~~~~~~~~~~~~~~~~~~~~~~~~~~~~~~~~~~~~~~~~~~~~~
**OsLAT7_OsPUT2** 1 ~~~~~~~~~~~~~~~~~~~~~~~~~~~~~~~~~~~~~~~~~~~~~~~~~~~~~~~~~~~~
OsLAT8 1 ~~~~~~~~~~~~~~~~~~~~~~~~~~~~~~~~~~~~~~~~ME..........TWLGC...
OsLAT9 1 MENGEIEGAADDGVPVPAPPNGRRYRPVGSSDRAVIQMTSMEPGSSSSTAVAAVSGITPQ

Sequence position 61 ........70........80........90.......100.......110.......120
AtAAP1 5 .....GGDDGLRRRGCSCTKDDFLPEESFQSMGNYLKALKETPSRFMDRIMTRSLDSDEI
**AtLAT1_AtPUT3_AtRMV1** 8 .....NLDSASQKPRISTENP.PPP.................PPHISI........GVTT
AtLAT3_AtPUT1 1 ~~~~~~~~~~~~~~~~~~~~~~~~~~~~~~~MGDYN..MNEFAYGNLY........DDDD
**AtLAT4_AtPUT2_AtPAR1** 1 ~~~~~~~~~~MQKRRIITVNP.SASIE....MSQYE..NNEVPYSSVG........AD..
AtPUT4 1 ~~~~~~~~~~~~~~~~~~~~~~~~~~~~~~~~~~~~~~~~~~~MAISEASK....SSHEL
AtPUT5 1 ~~~~~~~~~~~~~~~~~~~~~~~~~~~~~~~~~~~~~~~~~~~~~~~~~~~~~~~MGEEE
**OsLAT1_OsPUT1** 37 .....DTGQE............KPTVE....SAQPA..NGAAPMGEC.GTEY...RGLPD
OsLAT2 1 ~~~~~~MDQEIQLNQRPSQREQQQE.E.HGAT.......AAMPPQDDERQDHQAAAAVHG
OsLAT3 5 .....TMDQEIQLNQRPSPQRQQQAQE.HGGATAPA..PAPATPQDDEQQGHQAAVARHG
OsLAT4 1 ~~~~~~~~~~~~~~~~~~~~~~~MAAE.EAHRLADA..SATAPPKNAAAVDGVAVA.APA
**OsLAT5_OsPUT3_OsPAR1** 45 .....TAGQTLRPARGFTVEK.LRNTA....ITRAN..SACLPMEDCVGIKY...SSVNE
OsLAT6 1 ~~~~~~~~~~~~~~~~~~~~~~~~~~~~~~~~~~~~~~~~~~~~~~~~~~~~~~~~~~~~
**OsLAT7_OsPUT2** 1 ~~~~~~~~~~~~~~~~~~~~~~~~~~~~~~~~~~~~~~~~~~~~~~~~~~~~~~~~~~~~
OsLAT8 8 .........FYSKEKDLSDRGQWLNVGGEEPVVSY.Y......FSPATGTGT...RRRWR
OsLAT9 61 PPRNLTVDPSMQEDHTVSQGDSKLELFGFDSLVNILG......LKSMTGEQIQAPSSPRD

Sequence position 121 .......130.......140.......150.......160.......170.......180
AtAAP1 60 NEM.K..ARSGHEMKKTLTWWDLMWFGIGAVIGSGIFVLTGLEARNH..SGPAVV.LSYV
**AtLAT1_AtPUT3_AtRMV1** 37 GDP.ATSPARTVNQIKKITVLPLVFLIFYEVSG.GPF...GIEDSVKA.AGPLLAIVGFI
AtLAT3_AtPUT1 20 GDV.GGSSKEGNNSIQKVSMLPLVFLIFYEVSG.GPF...GAEGSVNA.AGPLLALLGFV
**AtLAT4_AtPUT2_AtPAR1** 34 .EV.PSSPPKATDKIRKVSMLPLVFLIFYEVSG.GPF...GVEDSVNA.AGPLLALLGFV
AtPUT4 14 PVT.TAESSGKKATAKKLTLIPLVFLIYFEVAG.GPF...GEEPAVQA.AGPLLAILGFL
AtPUT5 6 TIV.NDENSSKPKPSPKLTLLPLVFLIFYEVSG.GPF...GVEDSVKSGGGPLLALLGFL
**OsLAT1_OsPUT1** 70 GDA.G...GPMPSSARTVSMIPLIFLIFYEVSG.GPF...GIEDSVGA.AGPLLAIIGFL
OsLAT2 46 QGG.GGGATAERHHRSKLTLLPLVFLIYFEVAG.GPY...GAERAVRA.AGPLFALLGFL
OsLAT3 57 ....CGGATAERHHQTKLTLLPLVFFIYFEVAG.GPY...GAEQAVSA.AGPLFALLGFL
OsLAT4 34 AGG.....GHGRAPGNKLSLVPLIFLIFFEVAG.GPY...GAEPAVQS.AGPLFALLGFL
**OsLAT5_OsPUT3_OsPAR1** 90 GEE.R....KGGHGVPKVSIIPLIFLIFYEVSG.GPF...GIEDSVKA.AGPLLAIAGFL
OsLAT6 1 ~~~~~~~~~~~~~~~~~~~~~~~~~~~~~~~~~~~~~~~~~~~~~MGE.ARLLLAIIGFL
**OsLAT7_OsPUT2** 1 ~~M.TGACEAAPARRRGLTVLPLVALIFYDVSG.GPF...GIEDSVRAGGGALLPILGFL
OsLAT8 49 LEASAVGLGR.EETETKLDTMMGVFVPC.....................LQNILGIIYYI
OsLAT9 115 GEDVAITIGRPKETGPKFGTMMGVFVPC.....................LQNILGIIYYI

Sequence position 181 .......190.......200.......210.......220.......230.......240
AtAAP1 114 VSG.VSAML........SVFC......YT....EFAV.EIPVAGGSFAYLRVELGDFMAF
**AtLAT1_AtPUT3_AtRMV1** 91 VFPFIWSIP........EALI......TA....EMGT.MFPENGGYVVWVTLAMGPYWGF
AtLAT3_AtPUT1 74 IFPFIWCIP........EALI......TA....EMST.MFPINGGFVVWVSSALGTFWGF
**AtLAT4_AtPUT2_AtPAR1** 87 IFPFIWSIP........EALI......TA....EMGT.MYPENGGYVVWVSSALGPFWGF
AtPUT4 68 IFPFIWSIP........EALI......TA....ELST.AFPGNGGFVIWAHRAFGSFVGS
AtPUT5 61 IFPLIWSIP........EALV......TA....ELAT.SFPENGGYVVWISSAFGPFWGF
**OsLAT1_OsPUT1** 121 VLPVIWSIP........EALI......TA....ELGA.MFPENGGYVVWVASALGPYWGF
OsLAT2 100 AFPFAWGVP........VSLV......TA....ELAA.ALPGNGGFVVWADRAFGPLAGS
OsLAT3 108 AFPFAWGVP........VSLV......TA....ELAA.ALPGNGGFVVWADRAFGPLAGS
OsLAT4 84 VFPFIWAVP........ESLV......TA....ELAT.AMPGNGGFVLWADRAFGPFAGS
**OsLAT5_OsPUT3_OsPAR1** 140 LFALIWSVP........EALI......TA....EMGT.MFPENGGYVVWVSSALGPFWGF
OsLAT6 15 VLPVIWSIP........ETLI......TA....ELGA.MFPENGGYIVWVASALGPYWGF
**OsLAT7_OsPUT2** 54 VLPVLWSLP........EALV......TA....ELAS.AFPTNAGYVAWVSAAFGPAAAF
OsLAT8 87 RFTWIVGMGGVWQSLVLVAFCGSCTFLTTISLSAIATNGAMKGGGPYYLIGRALGPEVGV
OsLAT9 154 RFTWIVGMAGVWQSLVLVSFCGACTFLTGISLSAIATNGAMKGGGPYYLIGRALGPEVGV

Sequence position 241 .......250.......260.......270.......280.......290.......300
AtAAP1 154 IAAGNIILEYVVGGAAVARSWTSYFATLLNHKP...EDFRIIVHKLGEDYS.........
**AtLAT1_AtPUT3_AtRMV1** 132 QQGWVKWLSGVIDNALYPILFLDYLKSGIPI..............LGSG...........
AtLAT3_AtPUT1 115 QVGWMKWLCGVIDNALYPVLFLDYLKSAVPA..............LATG...........
**AtLAT4_AtPUT2_AtPAR1** 128 QQGWMKWLSGVIDNALYPVLFLDYLKSGVPA..............LGSG...........
AtPUT4 109 MMGSLKFLSGVINVASFPVLCVTYLDKLFPV..............LESG...........
AtPUT5 102 QEGFWKWFSGVMDNALYPVLFLDYLKHSFPV..............LDHV...........
**OsLAT1_OsPUT1** 162 QQGWMKWLSGVIDNALYPVLFLDYLKSGVPA..............LGGG...........
OsLAT2 141 LLGTWKYLSCVINLAAFPALVADYLGRVAPA..............VAVP...........
OsLAT3 149 LLGTWKYLSCVINLAAFPALVADYLGRVAPA..............VAVP...........
OsLAT4 125 LMGTWKYVSGAINGAAFPALCADYVARVAPA..............VSGG...........
**OsLAT5_OsPUT3_OsPAR1** 181 QQGWAKWLSGVIDNALYPVLFLDYVKSSIPA..............LGGG...........
OsLAT6 56 QQGWMKWLSGVIDNVLYPVLFLDYLKSGVPA..............LGRG...........
**OsLAT7_OsPUT2** 95 LVGFSKWASGTLDNALYPVLFLDYLRSGGGL..............VLSP...........
OsLAT8 147 SIGLCFFLGNAVAGAMYVLGAVETFLDAVPSAEFFQESVTVVTNTFVNGTAAGNATTIST
OsLAT9 214 SIGLCFFLGNAVAGSMYVLGAVETFLDAVPSAGFFKESVTVVNNTLVNGTATASTATIST

Sequence position 301 .......310.......320.......330.......340.......350.......360
AtAAP1 202 .HLDPIAVGVCAIICVLAVVGTKGSSRFNYIASIIHMVVI...LFVIIAGFTKADV..K.
**AtLAT1_AtPUT3_AtRMV1** 167 ...IPRVAAILVLTVALTYLNYRGLSIVGVAAVLLGVFSI..LPFVVMSFMSIPKLKPS.
AtLAT3_AtPUT1 150 ...LPRVASILILTLLLTYLNYRGLTIVGWTAVFMGVFSM..LPFAVMSLVSIPQLEPS.
**AtLAT4_AtPUT2_AtPAR1** 163 ...LPRVASILVLTILLTYLNYRGLTIVGWVAVLMGVFSI..LPFAVMGLISIPQLEPS.
AtPUT4 144 ...WPRNVCIFASTVVLSFLNYTGLAIVGYAAVVLGLVSL..SPFLVMSAMAIPKIKPH.
AtPUT5 137 ...AARVPALLVITFSLTYLNYRGLHIVGFSAVVLAVFSL..CPFVVMALLAVPNIRPK.
**OsLAT1_OsPUT1** 197 ...APRAFAVVGLTAVLTLLNYRGLTVVGWVAICLGVFSL..LPFFVMGLIALPKLRPA.
OsLAT2 176 .GSRARTGTVLGMTVFLSFLNLTGLSIVGWGAVALGFVSL..APFVLMTAMAAPRTRPR.
OsLAT3 184 .GSRARTGTVLGMTVFLSFLNLGGLSIVGWGAVALGFVSL..APFVLMTAMAAPRTRPR.
OsLAT4 160 ...GARVAAIVAFNVAISVLNYTGLSIVGWTAVALGVASL..SPFALMFGAALPKIRPR.
**OsLAT5_OsPUT3_OsPAR1** 216 ...LPRTLAVLILTVALTYMNYRGLTIVGWVAVFLGVFSL..LPFFVMGLIAIPRIEPS.
OsLAT6 91 ...ATRAFAVVGLMAVLTLLSYRGLTVVGWVAICLGVFSL..LPFFVMGLIALPRLRPA.
**OsLAT7_OsPUT2** 130 ...PARSLAVLALTAALTYLNFRGLHLVGLSALALTAFSL..SPFVALAVLAAPKIRPS.
OsLAT8 207 PNLHDLQVYGIIVTILLCFIVFGGVKIINKVAPAFLIPVLFSILCIYIGVFIAPRPNASK
OsLAT9 274 PSLHDLQVYGVIVTILLCFIVFGGVKIINKVAPAFLIPVLFSLLCIYLGVFIAPRHNAPK

Sequence position 361 .......370.......380.......390.......400.......410.......420
AtAAP1 255 ............NYSDFT.PYGVRGVFKSAAVLFFAYIG...........FDAVSTMAEE
**AtLAT1_AtPUT3_AtRMV1** 221 ............RWLVVSKKMKGVNWSLYLNTLFWNLNY...........WDSVSTLTGE
AtLAT3_AtPUT1 204 ............RWLVMD..LGNVNWNLYLNTLLWNLNY...........WDSVSTLAGE
**AtLAT4_AtPUT2_AtPAR1** 217 ............RWLVMD..LGNVNWNLYLNTLFWNLNY...........WDSISTLAGE
AtPUT4 198 ............RWGSLG..TKKKDWNLYFNTLFWNLNF...........WDNVSTLAGE
AtPUT5 191 ............RWLFVD..TQKINWRGYFNTMFWNLNY...........WDKASTLAGE
**OsLAT1_OsPUT1** 251 ............RWLVID..LHNVDWNLYLNTLFWNLNY...........WDSISTLAGE
OsLAT2 232 ............RWAARVKG.RKRDWRLFFNTLFWNLNY...........WDSASTMAGE
OsLAT3 240 ............RWAARVQVKGKRDWRLFFNTLFWNLNY...........WDSASTMAGE
OsLAT4 214 ............RWRATA...ADKDWKLFFNTLFWNLNY...........WDSASTMAGE
**OsLAT5_OsPUT3_OsPAR1** 270 ............RWLEMD..LGNVNWGLYLNTLFWNLNY...........WDSISTLAGE
OsLAT6 145 ............RWLVID..LHNVDWNLYLNTLFWNLNY...........WDSISTLAGE
**OsLAT7_OsPUT2** 184 ............RWLAVN..VAAVEPRAYFNSMFWNLNY...........WDKASTLAGE
OsLAT8 267 WITGLSITTLKDNWSSDYQRTNNAGVPDPNGA.......LLGLYFPAVTGIMAGSNRSAS
OsLAT9 334 GITGLSITTFKDNWGSEYQRTNNAGVPDPNGSIYWDFNALVGLFFPAVTGIMAGSNRSAS

Sequence position 421 .......430.......440.......450.......460.......470.......480
AtAAP1 291 TKNPGRDIPIGLVGSMVVTTVCYCLMAVT..LCLMQPYQQIDPDAPFSVAFSAVGWDWAK
**AtLAT1_AtPUT3_AtRMV1** 258 VENPSKTLPRALFYALLLVVFSYIFPVLTGTGAIAL.DQKLWTDGYFADIGKVIGGVWLG
AtLAT3_AtPUT1 239 VANPKKTLPKALCYGVIFVALSNFLPLLSGTGAIPL.DRELWTDGYLAEVAKAIGGGWLQ
**AtLAT4_AtPUT2_AtPAR1** 252 VENPNHTLPKALFYGVILVACSYIFPLLAGIGAIPL.EREKWTDGYFSDVAKALGGAWLR
AtPUT4 233 VDEPQKTFPLALLIAVIFTCVAYLIPLFAVTGAVSV.DQSRWENGFHAEAAEMIAGKWLK
AtPUT5 226 VDRPGKTFPKALFGAVLLVMGSYLIPLMAGTGALSSSTSGEWSDGYFAEVGMLIGGVWLK
**OsLAT1_OsPUT1** 286 VKNPGKTLPKALFYAVIFVVVAYLYPLLAGTGAVPL.DRGQWTDGYFADIAKLLGGAWLM
OsLAT2 268 VERPERTFPRALAVAVVLIAVSYLLPLMAAIGATDA.PPETWENGYLADAAGTWLSNSAL
OsLAT3 277 VERPERTFPRALAVAVVLIAVSYLLPLMAAVGATDA.PPEAWENGYLADAAATKLVRNLK
OsLAT4 248 VERPGRTFPRALLSAVAMTTLGYLLPLLAATGAIDA.APEDWGNGFFADAAGMIAGGWLK
**OsLAT5_OsPUT3_OsPAR1** 305 VENPKRTLPRALSYALVLVVGGYLYPLITCTAAVPV.VREFWTDGYFSDVARILGGFWLH
OsLAT6 180 VKNPGKTLPKALF~~~~~~~~~~~~~~~~~~~~~~~~~~~~~~~~~~~~~~~~~~~~~~~
**OsLAT7_OsPUT2** 219 VEEPRKTFPKAVFGAVGLVVGAYLIPLLAGTGALPSETAGEWTDGFFSVVGDRIGGPWLR
OsLAT8 320 LKDTQRSIPIGTLHATISTTMMYLLSVFL.FGALST.REGLLTDRLLC.....AAVAWPS
OsLAT9 394 LKDTQRSIPIGTLSATLTTTAMYLFSVLL.FGALAT.REELLTDRLLT.....ATVAWPA

Sequence position 481 .......490.......500.......510.......520.......530.......540
AtAAP1 349 Y.IVAFGALKGMTTVLLVGA....IGQARYMTHIARAHMMPPWLAQVNAK...TGTPINA
**AtLAT1_AtPUT3_AtRMV1** 317 W.WIQAAAATSNMGMFLAEMS....SDSFQLLGMAERGMLPEVFAK...RSR.YRTPWVG
AtLAT3_AtPUT1 298 L.WVQAAAATSNMGMFLAEMS....SDSFQLLGMAELGILPEIFAQ...RSR.YGTPLLG
**AtLAT4_AtPUT2_AtPAR1** 311 W.WVQAAAATSNMGMFIAEMS....SDSFQLLGMAERGMLPEFFAK...RSR.YGTPLLG
AtPUT4 292 I.WIEIGAVLSSIGLFEAQLS....SSAYQLEGMAELGFLPKFFGV...RSKWFNTPWVG
AtPUT5 286 G.WIQAAAAMSNLGLFEAEMS....SDAFQLLGMSEIGMLPAFFAQ...RSK.YGTPTIS
**OsLAT1_OsPUT1** 345 W.WVQSAAALSNMGMFVAEMS....SDSYQLLGMAERGMLPSFFAA...RSR.YGTPLAG
OsLAT2 327 .......SMAL~~~~~~~~~~~~~~~~~~~~~~~~~~~~~~~~~~~~~~~~~~~~~~~~~
OsLAT3 336 .......GPATSIPLYQNYNSLHHRRAVAQVLDGGRRGALLRRVVRSAAEQRRVPAPRHG
OsLAT4 307 Y.WIEVGAVLSTIGLYSATLS....SAAFQLLGMADLGLLPRAFA.LRAP..VFDTPWVG
**OsLAT5_OsPUT3_OsPAR1** 364 S.WLQAAAALSNMGNFVTEMS....SDSYQLLGMAERGMLPEFFAK...RSR.YGTPLIG
OsLAT6 193 ~~~~~~~~~~~~~~~~~~~~~~~~~~~~~~~~~~~~~~~~~~~~~~~~~~~~~~~~~~~~
**OsLAT7_OsPUT2** 279 V.WIQAAAAMSNMGLFEAEMS....GDSFQLLGMAEMGMIPAIFAR...RSR.HGTPTYS
OsLAT8 373 PAVVYA......................................................
OsLAT9 447 PAVIYIGIILSTLGAALQSLT....GAPRLLAAIANDDILPVLNYF...KVSEGAEPHSA

Sequence position 541 .......550.......560.......570.......580.......590.......600
AtAAP1 401 TVVMLAATALIAFFTKLKILADLLSVSTLFIFMFVAVALLVRRYYVTGETSTRDRNKFLV
**AtLAT1_AtPUT3_AtRMV1** 368 ILFSASGVIILSWLSFQEIV.................................AAENL..
AtLAT3_AtPUT1 349 ILFSASGVLLLSGLSFQEII.................................AAENL..
**AtLAT4_AtPUT2_AtPAR1** 362 ILFSASGVVLLSWLSFQEIV.................................AAENL..
AtPUT4 344 ILISALMSLGLSYMNFTDII.................................SSANF..
AtPUT5 337 ILCSATGVIFLSWMSFQEII.................................EFLNF..
**OsLAT1_OsPUT1** 396 ILFSASGVLLLSMMSFQEIV.................................AAENF..
OsLAT2 331 ~~~~~~~~~~~~~~~~~~~~~~~~~~~~~~~~~~~~~~~~~~~~~~~~~~~~~~~~~~~~
OsLAT3 389 GAGPPPLRLR..PPRPRTIRHP.............................VGRRRRL..
OsLAT4 359 ILATAAITLAMSFTSFDTIV.................................ASANF..
**OsLAT5_OsPUT3_OsPAR1** 415 IMFSAFGVVLLSWMSFQEII.................................AAENY..
OsLAT6 193 ~~~~~~~~~~~~~~~~~~~~~~~~~~~~~~~~~~~~~~~~~~~~~~~~~~~~~~~~~~~~
**OsLAT7_OsPUT2** 330 ILCSATGVVILSFMSFQEIV.................................EFLNF..
OsLAT8 379 ............................................................
OsLAT9 500 TLFTAFICICCVVIGNLDLITPTITM..FFLLCYAGVNLSCFLLDLLDAPSWRPRWKFHH

Sequence position 601 .......610.......620.......630.......640.......650.......660
AtAAP1 461 F....LGLILA...............SSTATAVYWALEE....EGWIGYCITVPIWFLST
**AtLAT1_AtPUT3_AtRMV1** 393 ...............................................LYCFGMVLEFITF
AtLAT3_AtPUT1 374 ...............................................LYCGGMILEFIAF
**AtLAT4_AtPUT2_AtPAR1** 387 ...............................................LYCVGMILEFIAF
AtPUT4 369 ...............................................LYTLGMFLEFASF
AtPUT5 362 ...............................................LYALGMLLEFAAF
**OsLAT1_OsPUT1** 421 ...............................................LYCFGMLLEFVAF
OsLAT2 331 ~~~~~~~~~~~~~~~~~~~~~~~~~~~~~~~~~~~~~~~~~~~~~~~~~~~~~~~~~~~~
OsLAT3 416 ...............................................RRRLGRRLLPRLR
OsLAT4 384 ...............................................LYSLGMLLEFAAF
**OsLAT5_OsPUT3_OsPAR1** 440 ...............................................LYCFGMILEFIAF
OsLAT6 193 ~~~~~~~~~~~~~~~~~~~~~~~~~~~~~~~~~~~~~~~~~~~~~~~~~~~~~~~~~~~~
**OsLAT7_OsPUT2** 355 ...............................................LYGLGMLAVFAAF
OsLAT8 379 ............VIMFMISWTFTVVSLALASLIYYYVSLKGKAGDW.GDGFKSAYFQLAL
OsLAT9 558 WSLSLVGALLCVVIMFLISWSFTVVSLALASLIYYYVSLKGKAGDW.GDGFKSAYFQLAL

Sequence position 661 .......670.......680.......690.......700.......710.......720
AtAAP1 498 VAMKFLVPQARAP.KIWGVPL......VP........WLPSASIAIN.............
**AtLAT1_AtPUT3_AtRMV1** 406 VRLRMKYPAASRP...FKIPV......GV.LGSVLMCIPPTVLIGVIMAFTNLKVALVSL
AtLAT3_AtPUT1 387 VRLRKKHPAASRP...YKIPV......GT.VGSILICVPPIVLICLVIVLSTIKVALVSF
**AtLAT4_AtPUT2_AtPAR1** 400 VRMRMKHPAASRP...YKIPI......GT.TGSILMCIPPTILICAVVALSSLKVAAVSI
AtPUT4 382 IWLRRKLPQLKRP...YRVPL......KI.PGLVVMCLIPSAFLVLILVFATKIVYLICG
AtPUT5 375 VKLRIKKPDLHRP...YRVPL......NT.FGVSMLCLPPSLLVILVMVLAAPKTFLISG
**OsLAT1_OsPUT1** 434 ILHRVRRPDAARP...YRVPL......GT.AGCVAMLVPPTALIAVVLALSTLKVAVVSL
OsLAT2 331 ~~~~~~~~~~~~~~~~~~~~~~~~~~~~~~~~~~~~~~~~~~~~~~~~~~~~~~~~~~~~
OsLAT3 429 RRRRHRQPALQPR...RAARV......RRLPPAPREGGEPLLAQAP.LPRPAAAPRARRH
OsLAT4 397 VRLRARLPAMPRP...YAVPL......RGLPAAAALCAVPSAFLVFVMAIAGWKVYAISA
**OsLAT5_OsPUT3_OsPAR1** 453 IKLRVVHPNASRP...YKIPL......GT.IGAVLMIIPPTILIVVVMMLASFKVMVVSI
OsLAT6 193 ~~~~~~~~~~~~~~~~~~~~~~~~~~~~~~~~~~~~~~~~~~~~~~~~~~~~~~~~~~~~
**OsLAT7_OsPUT2** 368 VKLRVKDPDLPRP...YRIPV......GA.AGAAAMCVPPVVLITTVMCLASARTLVVSA
OsLAT8 426 RSLRSMGANQVHPKNWYPIPLIFCRPWGKLP.EDVPCHPKLADFANC.............
OsLAT9 617 RSLRSLGANQVHPKNWYPIPLIFCRPWGKLP.ENVPCHPKLADFANC.............

Sequence position 721 .......730.......740.......750.......760.......770.......780
AtAAP1 530 .........IFLLGSIDTKSFVRFAIWTGILLIYYVLFGLHATYDTAKATLKEKQALQKA
**AtLAT1_AtPUT3_AtRMV1** 456 AAIVIGLVLQPCLKQVEKKGWLKFSTSSHLPNLM.............E~~~~~~~~~~~~
AtLAT3_AtPUT1 437 VMVVIGFLMKPCLNHMDGKKWVKFSVCSDLAEFQ.............K...ENLDC....
**AtLAT4_AtPUT2_AtPAR1** 450 VMMIIGFLIHPLLNHMDRKRWVKFSISSDLPDLQ.............Q...QTREY....
AtPUT4 432 VMTIGAIGWYFLINYFRKTKIFEFNEVIDDLDNN.............V..NGEHPKVDDH
AtPUT5 425 VIIVLGFCLYPFLTLVKEKQWARFIPEETRPVSG.............V...SSESQLDEE
**OsLAT1_OsPUT1** 484 GAVAMGLVLQPALRFVEKKRWLRFSVNPDLPEIG.............V...IRPPA....
OsLAT2 331 ~~~~~~~~~~~~~~~~~~~~~~~~~~~~~~~~~~~~~~~~~~~~~~~~~~~~~~~~~~~~
OsLAT3 479 VPRAVGV........PGVRGRRRRVEGLRRRRRA.............H...GPRRRLARR
OsLAT4 448 VFTAAGVAVYYLMDLCKARGWLTFS.AAAADRGG.............S...GGDA.MMYR
**OsLAT5_OsPUT3_OsPAR1** 503 MAMLVGFVLQPALVYVEKRRWLKFSISAELPDLP.............Y...SNVEE....
OsLAT6 193 ~~~~~~~~~~~~~~~~~~~~~~~~~~~~~~~~~~~~~~~~~~~~~~~~~~~~~~~~~~~~
**OsLAT7_OsPUT2** 418 AVAVAGVAMYYGVEHMKATGCVEFLTPVPPDSLR.............GSSSSSSSSAASD
OsLAT8 472 ...............MKKKG.RGMSIFVSIIDGDY.........................
OsLAT9 663 ...............MKKKG.RGMSIFVSIIDGDY.........................

Sequence position 781 .......790.......800.......810.......820.......830.......840
AtAAP1 581 EEGGVV......................ADNSCSAT~~~~~~~~~~~~~~~~~~~~~~~~
**AtLAT1_AtPUT3_AtRMV1** 491 ~~~~~~~~~~~~~~~~~~~~~~~~~~~~~~~~~~~~~~~~~~~~~~~~~~~~~~~~~~~~
AtLAT3_AtPUT1 477 ...EESLLR~~~~~~~~~~~~~~~~~~~~~~~~~~~~~~~~~~~~~~~~~~~~~~~~~~~
**AtLAT4_AtPUT2_AtPAR1** 490 ...EETLIR~~~~~~~~~~~~~~~~~~~~~~~~~~~~~~~~~~~~~~~~~~~~~~~~~~~
AtPUT4 477 NS~~~~~~~~~~~~~~~~~~~~~~~~~~~~~~~~~~~~~~~~~~~~~~~~~~~~~~~~~~
AtPUT5 469 HGDESAASLLP~~~~~~~~~~~~~~~~~~~~~~~~~~~~~~~~~~~~~~~~~~~~~~~~~
**OsLAT1_OsPUT1** 524 ...APDEPLVP~~~~~~~~~~~~~~~~~~~~~~~~~~~~~~~~~~~~~~~~~~~~~~~~~
OsLAT2 331 ~~~~~~~~~~~~~~~~~~~~~~~~~~~~~~~~~~~~~~~~~~~~~~~~~~~~~~~~~~~~
OsLAT3 515 HEGVQV.QEVAQ.....VQHRGCRRPSSATTRCSSSSCW~~~~~~~~~~~~~~~~~~~~~
OsLAT4 490 RQGSTA.SEVV~~~~~~~~~~~~~~~~~~~~~~~~~~~~~~~~~~~~~~~~~~~~~~~~~
**OsLAT5_OsPUT3_OsPAR1** 543 ...DSTIPLVC~~~~~~~~~~~~~~~~~~~~~~~~~~~~~~~~~~~~~~~~~~~~~~~~~
OsLAT6 193 ~~~~~~~~~~~~~~~~~~~~~~~~~~~~~~~~~~~~~~~~~~~~~~~~~~~~~~~~~~~~
**OsLAT7_OsPUT2** 465 NGGDDDVEDVCALLLAAGEHAGEGVSVS.......KENY~~~~~~~~~~~~~~~~~~~~~
OsLAT8 491 ......................HESAEDAKTACRQLSAYIDYRRCEGVAEIIVAPSTSIG
OsLAT9 682 ......................HELAEDAKTACRQLDTYIEYKRCEGVAEIIVAPSMSEG

Sequence position 841 .......850.......860.......870.......880.......890.......900
AtAAP1 595 ~~~~~~~~~~~~~~~~~~~~~~~~~~~~~~~~~~~~~~~~~~~~~~~~~~~~~~~~~~~~
**AtLAT1_AtPUT3_AtRMV1** 491 ~~~~~~~~~~~~~~~~~~~~~~~~~~~~~~~~~~~~~~~~~~~~~~~~~~~~~~~~~~~~
AtLAT3_AtPUT1 483 ~~~~~~~~~~~~~~~~~~~~~~~~~~~~~~~~~~~~~~~~~~~~~~~~~~~~~~~~~~~~
**AtLAT4_AtPUT2_AtPAR1** 496 ~~~~~~~~~~~~~~~~~~~~~~~~~~~~~~~~~~~~~~~~~~~~~~~~~~~~~~~~~~~~
AtPUT4 479 ~~~~~~~~~~~~~~~~~~~~~~~~~~~~~~~~~~~~~~~~~~~~~~~~~~~~~~~~~~~~
AtPUT5 480 ~~~~~~~~~~~~~~~~~~~~~~~~~~~~~~~~~~~~~~~~~~~~~~~~~~~~~~~~~~~~
**OsLAT1_OsPUT1** 532 ~~~~~~~~~~~~~~~~~~~~~~~~~~~~~~~~~~~~~~~~~~~~~~~~~~~~~~~~~~~~
OsLAT2 331 ~~~~~~~~~~~~~~~~~~~~~~~~~~~~~~~~~~~~~~~~~~~~~~~~~~~~~~~~~~~~
OsLAT3 548 ~~~~~~~~~~~~~~~~~~~~~~~~~~~~~~~~~~~~~~~~~~~~~~~~~~~~~~~~~~~~
OsLAT4 500 ~~~~~~~~~~~~~~~~~~~~~~~~~~~~~~~~~~~~~~~~~~~~~~~~~~~~~~~~~~~~
**OsLAT5_OsPUT3_OsPAR1** 551 ~~~~~~~~~~~~~~~~~~~~~~~~~~~~~~~~~~~~~~~~~~~~~~~~~~~~~~~~~~~~
OsLAT6 193 ~~~~~~~~~~~~~~~~~~~~~~~~~~~~~~~~~~~~~~~~~~~~~~~~~~~~~~~~~~~~
**OsLAT7_OsPUT2** 497 ~~~~~~~~~~~~~~~~~~~~~~~~~~~~~~~~~~~~~~~~~~~~~~~~~~~~~~~~~~~~
OsLAT8 529 FRSIVQTMGLGNLKPNIVVMRYPEIWRRENLTQIPSTFVSIINDCITANKAVVIVKGLDE
OsLAT9 720 FRSIVQTMGLGNLKPNIIVMRYPEIWRRENLIQIPSTFVSIINDCIIANKAVVIVKGLDE

Sequence position 901 .......910.......920.......930.......940.......950.......960
AtAAP1 595 ~~~~~~~~~~~~~~~~~~~~~~~~~~~~~~~~~~~~~~~~~~~~~~~~~~~~~~~~~~~~
**AtLAT1_AtPUT3_AtRMV1** 491 ~~~~~~~~~~~~~~~~~~~~~~~~~~~~~~~~~~~~~~~~~~~~~~~~~~~~~~~~~~~~
AtLAT3_AtPUT1 483 ~~~~~~~~~~~~~~~~~~~~~~~~~~~~~~~~~~~~~~~~~~~~~~~~~~~~~~~~~~~~
**AtLAT4_AtPUT2_AtPAR1** 496 ~~~~~~~~~~~~~~~~~~~~~~~~~~~~~~~~~~~~~~~~~~~~~~~~~~~~~~~~~~~~
AtPUT4 479 ~~~~~~~~~~~~~~~~~~~~~~~~~~~~~~~~~~~~~~~~~~~~~~~~~~~~~~~~~~~~
AtPUT5 480 ~~~~~~~~~~~~~~~~~~~~~~~~~~~~~~~~~~~~~~~~~~~~~~~~~~~~~~~~~~~~
**OsLAT1_OsPUT1** 532 ~~~~~~~~~~~~~~~~~~~~~~~~~~~~~~~~~~~~~~~~~~~~~~~~~~~~~~~~~~~~
OsLAT2 331 ~~~~~~~~~~~~~~~~~~~~~~~~~~~~~~~~~~~~~~~~~~~~~~~~~~~~~~~~~~~~
OsLAT3 548 ~~~~~~~~~~~~~~~~~~~~~~~~~~~~~~~~~~~~~~~~~~~~~~~~~~~~~~~~~~~~
OsLAT4 500 ~~~~~~~~~~~~~~~~~~~~~~~~~~~~~~~~~~~~~~~~~~~~~~~~~~~~~~~~~~~~
**OsLAT5_OsPUT3_OsPAR1** 551 ~~~~~~~~~~~~~~~~~~~~~~~~~~~~~~~~~~~~~~~~~~~~~~~~~~~~~~~~~~~~
OsLAT6 193 ~~~~~~~~~~~~~~~~~~~~~~~~~~~~~~~~~~~~~~~~~~~~~~~~~~~~~~~~~~~~
**OsLAT7_OsPUT2** 497 ~~~~~~~~~~~~~~~~~~~~~~~~~~~~~~~~~~~~~~~~~~~~~~~~~~~~~~~~~~~~
OsLAT8 589 WPNEYQRQYGTIDLYWIVRDGGLMLLLSQLLLTKESFESCKIQVFCIAEEDTEAEELKAD
OsLAT9 780 WPNEYQRQYGTIDLYWIVRDGGLMLLLSQLLLTKETFESCKIQVFCIAEEDTDAEELKAD

Sequence position 961 .......970.......980.......990......1000......1010......1020
AtAAP1 595 ~~~~~~~~~~~~~~~~~~~~~~~~~~~~~~~~~~~~~~~~~~~~~~~~~~~~~~~~~~~~
**AtLAT1_AtPUT3_AtRMV1** 491 ~~~~~~~~~~~~~~~~~~~~~~~~~~~~~~~~~~~~~~~~~~~~~~~~~~~~~~~~~~~~
AtLAT3_AtPUT1 483 ~~~~~~~~~~~~~~~~~~~~~~~~~~~~~~~~~~~~~~~~~~~~~~~~~~~~~~~~~~~~
**AtLAT4_AtPUT2_AtPAR1** 496 ~~~~~~~~~~~~~~~~~~~~~~~~~~~~~~~~~~~~~~~~~~~~~~~~~~~~~~~~~~~~
AtPUT4 479 ~~~~~~~~~~~~~~~~~~~~~~~~~~~~~~~~~~~~~~~~~~~~~~~~~~~~~~~~~~~~
AtPUT5 480 ~~~~~~~~~~~~~~~~~~~~~~~~~~~~~~~~~~~~~~~~~~~~~~~~~~~~~~~~~~~~
**OsLAT1_OsPUT1** 532 ~~~~~~~~~~~~~~~~~~~~~~~~~~~~~~~~~~~~~~~~~~~~~~~~~~~~~~~~~~~~
OsLAT2 331 ~~~~~~~~~~~~~~~~~~~~~~~~~~~~~~~~~~~~~~~~~~~~~~~~~~~~~~~~~~~~
OsLAT3 548 ~~~~~~~~~~~~~~~~~~~~~~~~~~~~~~~~~~~~~~~~~~~~~~~~~~~~~~~~~~~~
OsLAT4 500 ~~~~~~~~~~~~~~~~~~~~~~~~~~~~~~~~~~~~~~~~~~~~~~~~~~~~~~~~~~~~
**OsLAT5_OsPUT3_OsPAR1** 551 ~~~~~~~~~~~~~~~~~~~~~~~~~~~~~~~~~~~~~~~~~~~~~~~~~~~~~~~~~~~~
OsLAT6 193 ~~~~~~~~~~~~~~~~~~~~~~~~~~~~~~~~~~~~~~~~~~~~~~~~~~~~~~~~~~~~
**OsLAT7_OsPUT2** 497 ~~~~~~~~~~~~~~~~~~~~~~~~~~~~~~~~~~~~~~~~~~~~~~~~~~~~~~~~~~~~
OsLAT8 649 VKKFLYDLRMQADVIVVTVKSWEADPD..RSGGSKKDDPEVYRSAQSRIRTYISQLKEAA
OsLAT9 840 VKKFLYDLRMHAEVIVVTMKSWEPHMESSSSGAPQDDSQEAYTSAQRRISTYLSEMKETA

Sequence position 1021 ......1030......1040......1050......1060......1070......1080
AtAAP1 595 ~~~~~~~~~~~~~~~~~~~~~~~~~~~~~~~~~~~~~~~~~~~~~~~~~~~~~~~~~~~~
**AtLAT1_AtPUT3_AtRMV1** 491 ~~~~~~~~~~~~~~~~~~~~~~~~~~~~~~~~~~~~~~~~~~~~~~~~~~~~~~~~~~~~
AtLAT3_AtPUT1 483 ~~~~~~~~~~~~~~~~~~~~~~~~~~~~~~~~~~~~~~~~~~~~~~~~~~~~~~~~~~~~
**AtLAT4_AtPUT2_AtPAR1** 496 ~~~~~~~~~~~~~~~~~~~~~~~~~~~~~~~~~~~~~~~~~~~~~~~~~~~~~~~~~~~~
AtPUT4 479 ~~~~~~~~~~~~~~~~~~~~~~~~~~~~~~~~~~~~~~~~~~~~~~~~~~~~~~~~~~~~
AtPUT5 480 ~~~~~~~~~~~~~~~~~~~~~~~~~~~~~~~~~~~~~~~~~~~~~~~~~~~~~~~~~~~~
**OsLAT1_OsPUT1** 532 ~~~~~~~~~~~~~~~~~~~~~~~~~~~~~~~~~~~~~~~~~~~~~~~~~~~~~~~~~~~~
OsLAT2 331 ~~~~~~~~~~~~~~~~~~~~~~~~~~~~~~~~~~~~~~~~~~~~~~~~~~~~~~~~~~~~
OsLAT3 548 ~~~~~~~~~~~~~~~~~~~~~~~~~~~~~~~~~~~~~~~~~~~~~~~~~~~~~~~~~~~~
OsLAT4 500 ~~~~~~~~~~~~~~~~~~~~~~~~~~~~~~~~~~~~~~~~~~~~~~~~~~~~~~~~~~~~
**OsLAT5_OsPUT3_OsPAR1** 551 ~~~~~~~~~~~~~~~~~~~~~~~~~~~~~~~~~~~~~~~~~~~~~~~~~~~~~~~~~~~~
OsLAT6 193 ~~~~~~~~~~~~~~~~~~~~~~~~~~~~~~~~~~~~~~~~~~~~~~~~~~~~~~~~~~~~
**OsLAT7_OsPUT2** 497 ~~~~~~~~~~~~~~~~~~~~~~~~~~~~~~~~~~~~~~~~~~~~~~~~~~~~~~~~~~~~
OsLAT8 707 ERERRPLMEGGKQVVVDEQKVEKFLYTMLKLNATILRHSRMAVVVLVSLPPPPLNHLAYC
OsLAT9 900 QREGHPLMEDGKQVVVNEQKIEKFLYTMFKLNSTILRYSRMAAVVLVSLPPPPLNHPAYF

Sequence position 1081 ......1090......1100......1110
AtAAP1 595 ~~~~~~~~~~~~~~~~~~~~~~~~~~~~~~
**AtLAT1_AtPUT3_AtRMV1** 491 ~~~~~~~~~~~~~~~~~~~~~~~~~~~~~~
AtLAT3_AtPUT1 483 ~~~~~~~~~~~~~~~~~~~~~~~~~~~~~~
**AtLAT4_AtPUT2_AtPAR1** 496 ~~~~~~~~~~~~~~~~~~~~~~~~~~~~~~
AtPUT4 479 ~~~~~~~~~~~~~~~~~~~~~~~~~~~~~~
AtPUT5 480 ~~~~~~~~~~~~~~~~~~~~~~~~~~~~~~
**OsLAT1_OsPUT1** 532 ~~~~~~~~~~~~~~~~~~~~~~~~~~~~~~
OsLAT2 331 ~~~~~~~~~~~~~~~~~~~~~~~~~~~~~~
OsLAT3 548 ~~~~~~~~~~~~~~~~~~~~~~~~~~~~~~
OsLAT4 500 ~~~~~~~~~~~~~~~~~~~~~~~~~~~~~~
**OsLAT5_OsPUT3_OsPAR1** 551 ~~~~~~~~~~~~~~~~~~~~~~~~~~~~~~
OsLAT6 193 ~~~~~~~~~~~~~~~~~~~~~~~~~~~~~~
**OsLAT7_OsPUT2** 497 ~~~~~~~~~~~~~~~~~~~~~~~~~~~~~~
OsLAT8 767 YMEYMDLLVENIPRILIVRGYRRDVVTLFT
OsLAT9 960 YMEYMDLLVENVPRMLIVRGYRRDVVTFFT
