# Supplementary material for: Methylviologen resistance in loss-of-function mutants of the polyamine transporter gene OsLAT5
Source: PLoS One. 2026 Apr 16;21(4):e0346828. doi: 10.1371/journal.pone.0346828 (PMC13086316; doi:10.1371/journal.pone.0346828)

**Supporting Information S3. Organ-specific mRNA level for (A) *OsLAT5*/*OsPUT3*/*OsPAR1*, (B) *OsLAT1*/*OsPUT1* and (C) *OsLAT7*/*OsPUT2*** [37]. FPKM: Fragments Per Kilobase of transcript per Million mapped reads.

**B**


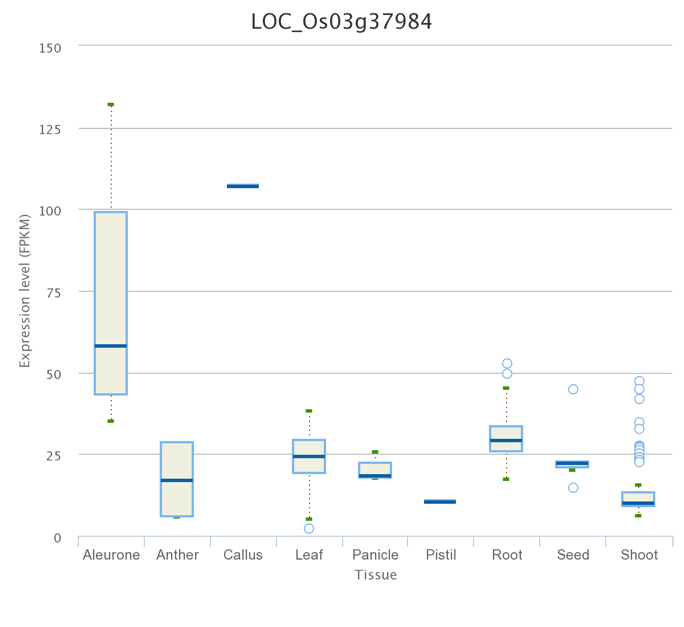

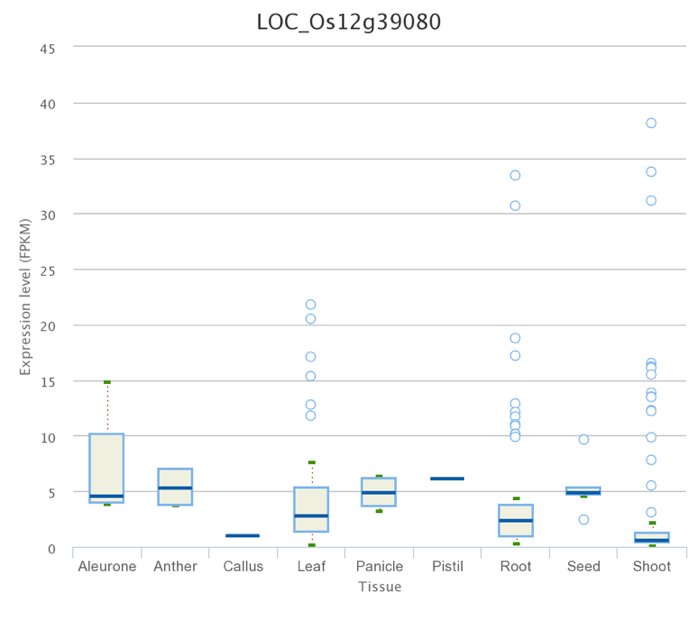


**A**

**C**


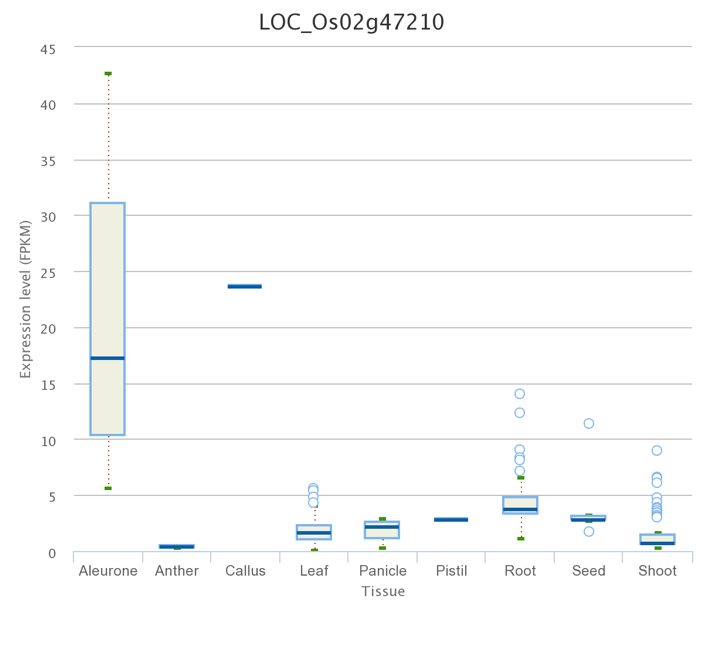

Supplement: S3 File — FPKM: Fragments Per Kilobase of transcript per Million mapped reads. (DOCX) [file pone.0346828.s003.docx]
