# Supplementary material for: Methylviologen resistance in loss-of-function mutants of the polyamine transporter gene OsLAT5
Source: PLoS One. 2026 Apr 16;21(4):e0346828. doi: 10.1371/journal.pone.0346828 (PMC13086316; doi:10.1371/journal.pone.0346828)
Supplement: S4 File — The following parts of the T-DNA are annotated with colors: RIGHT AND LEFT BORDERS, U6 PROMOTER, GLYCINE tRNA, gRNA TARGET SITES from the S1 Table, MAIZE UBIQUITIN PROMOTER, CAS9 coding sequence, NOPALINE SYNTHASE (NOS) TERMINATOR, CAULIFLOWER MOSAIC VIRUS 35S PROMOTER, HYGROMYCIN PHOSPHOTRANSFERASE. (DOCX) [file pone.0346828.s004.docx]

**Supporting Information S4. Vector sequences used for editing individual *OsLAT* genes.** The following parts of the T-DNA are annotated with colors: RIGHT AND LEFT BORDERS, *U6* PROMOTER, GLYCINE tRNA, gRNA TARGET SITES from the S1 Table, MAIZE *UBIQUITIN* PROMOTER, CAS9 coding sequence, *NOPALINE SYNTHASE* (NOS) TERMINATOR, CAULIFLOWER MOSAIC VIRUS 35S PROMOTER, ***HYGROMYCIN PHOSPHOTRANSFERASE***.

GTTTACCCGCCAATATATCCTGTCAAACACTGATAGTTTAAACTGAAGGCGGGAAACGACAATCTGATCCAAGCTCAAGCTGCTCTAGCATTCGCCATTCAGGCTGCGCAACTGTTGGGAAGGGCGATCGGTGCGGGCCTCTTCGCTATTACGCCAGCTGGCGAAAGGGGGATGTGCTGCAAGGCGATTAAGTTGGGTAACGCCAGGGTTTTCCCAGTCACGACGTTGTAAAACGACGGCCAGTGCCAAGCTAGAGACGGGGATCACAAGTTTGTACAAAAAACCACCATGGGAACCAATTCAGTCGACTGGATCCAAGCTTAAGAACGAACTAAGCCGGACAAAAAAAGGAGCACATATACAAACCGGTTTTATTCATGAATGGTCACGATGGATGATGGGGCTCAGACTTGAGCTACGAGGCCGCAGGCGAGAGAAGCCTAGTGTGCTCTCTGCTTGTTTGGGCCGTAACGGAGGATACGGCCGACGAGCGTGTACTACCGCGCGGGATGCCGCTGGGCGCTGCGGGGGCCGTTGGATGGGGATCGGTGGGTCGCGGGAGCGTTGAGGGGAGACAGGTTTAGTACCACCTCGCCTACCGAACAATGAAGAACCCACCTTATAACCCCGCGCGCTGCCGCTTGTGTTGAACAAAGCACCAGTGGTCTAGTGGTAGAATAGTACCCTGCCACGGTACAGACCCGGGTTCGATTCCCGGCTGGTGCAGNNNNNNNNNNNNNNNNNNGTTTTAGAGCTAGAAATAGCAAGTTAAAATAAGGCTAGTCCGTTATCAACTTGAAAAAGTGGCACCGAGTCGGTGCGGGAGCACATGAGGATCACCCATGTGCCACGAGCGACATGAGGATCACCCATGTCGCTCGTGTTCCCAACAAAGCACCAGTGGTCTAGTGGTAGAATAGTACCCTGCCACGGTACAGACCCGGGTTCGATTCCCGGCTGGTGCAGNNNNNNNNNNNNNNNNNNNGTTTTAGAGCTAGAAATAGCAAGTTAAAATAAGGCTAGTCCGTTATCAACTTGAAAAAGTGGCACCGAGTCGGTGCGGGAGCACATGAGGATCACCCATGTGCCACGAGCGACATGAGGATCACCCATGTCGCTCGTGTTCCCTTTTTTTGTCCCTTCGAAGGGCAATTCTGCAGATATCCATCACACTGGCGGCCGGTTTAAGCTTGAATTCACTCGAGATATCTAGTTCTTGTACAAAGTGGTGAAGCTTCTGCAGTGCAGCGTGACCCGGTCGTGCCCCTCTCTAGAGATAATGAGCATTGCATGTCTAAGTTATAAAAAATTACCACATATTTTTTTTGTCACACTTGTTTGAAGTGCAGTTTATCTATCTTTATACATATATTTAAACTTTACTCTACGAATAATATAATCTATAGTACTACAATAATATCAGTGTTTTAGAGAATCATATAAATGAACAGTTAGACATGGTCTAAAGGACAATTGAGTATTTTGACAACAGGACTCTACAGTTTTATCTTTTTAGTGTGCATGTGTTCTCCTTTTTTTTTGCAAATAGCTTCACCTATATAATACTTCATCCATTTTATTAGTACATCCATTTAGGGTTTAGGGTTAATGGTTTTTATAGACTAATTTTTTTAGTACATCTATTTTATTCTATTTTAGCCTCTAAATTAAGAAAACTAAAACTCTATTTTAGTTTTTTTATTTAATAATTTAGATATAAAATAGAATAAAATAAAGTGACTAAAAATTAAACAAATACCCTTTAAGAAATTAAAAAAACTAAGGAAACATTTTTCTTGTTTCGAGTAGATAATGCCAGCCTGTTAAACGCCGTCGACGAGTCTAACGGACACCAACCAGCGAACCAGCAGCGTCGCGTCGGGCCAAGCGAAGCAGACGGCACGGCATCTCTGTCGCTGCCTCTGGACCCCTCTCGAGAGTTCCGCTCCACCGTTGGACTTGCTCCGCTGTCGGCATCCAGAAATTGCGTGGCGGAGCGGCAGACGTGAGCCGGCACGGCAGGCGGCCTCCTCCTCCTCTCACGGCACGGCAGCTACGGGGGATTCCTTTCCCACCGCTCCTTCGCTTTCCCTTCCTCGCCCGCCGTAATAAATAGACACCCCCTCCACACCCTCTTTCCCCAACCTCGTGTTGTTCGGAGCGCACACACACACAACCAGATCTCCCCCAAATCCACCCGTCGGCACCTCCGCTTCAAGGTACGCCGCTCGTCCTCCCCCCCCCCCCCTCTCTACCTTCTCTAGATCGGCGTTCCGGTCCATGGTTAGGGCCCGGTAGTTCTACTTCTGTTCATGTTTGTGTTAGATCCGTGTTTGTGTTAGATCCGTGCTGCTAGCGTTCGTACACGGATGCGACCTGTACGTCAGACACGTTCTGATTGCTAACTTGCCAGTGTTTCTCTTTGGGGAATCCTGGGATGGCTCTAGCCGTTCCGCAGACGGGATCGATTTCATGATTTTTTTTGTTTCGTTGCATAGGGTTTGGTTTGCCCTTTTCCTTTATTTCAATATATGCCGTGCACTTGTTTGTCGGGTCATCTTTTCATGCTTTTTTTTGTCTTGGTTGTGATGATGTGGTGTGGTTGGGCGGTCGTTCATTCGTTCTAGATCGGAGTAGAATACTGTTTCAAACTACCTGGTGTATTTATTAATTTTGGAACTGTATGTGTGTGTCATACATCTTCATAGTTACGAGTTTAAGATGGATGGAAATATCGATCTAGGATAGGTATACATGTTGATGTGGGTTTTACTGATGCATATACATGATGGCATATGCAGCATCTATTCATATGCTCTAACCTTGAGTACCTATCTATTATAATAAACAAGTATGTTTTATAATTATTTTGATCTTGATATACTTGGATGATGGCATATGCAGCAGCTATATGTGGATTTTTTTAGCCCTGCCTTCATACGCTATTTATTTGCTTGGTACTGTTTCTTTTGTCGATGCTCACCCTGTTGTTTGGTGTTACTTCTGCAGGTCGACTCTAGAGGATCTGGAATTCCCGGGTACCGGATCCAAATCCGAATCCGGCCATGGACTATAAGGATCACGATGGCGACTACAAGGATCATGACATTGACTATAAGGATGACGACGATAAGATGGCACCTAAGAAGAAAAGGAAAGTCGGCATTCATGGCGTTCCGGCAGCCGACAAAAAGTATAGCATCGGCCTCGATATTGGGACAAACTCTGTGGGCTGGGCGGTAATTACCGACGAGTACAAGGTGCCTAGTAAGAAATTTAAAGTGCTCGGAAACACTGACAGGCACTCTATAAAGAAGAACCTGATCGGGGCACTGCTTTTCGACTCCGGAGAGACGGCGGAGGCGACGCGTCTCAAGCGTACCGCGCGCCGCAGGTACACAAGAAGGAAGAATAGGATCTGCTACTTGCAGGAAATCTTCAGTAACGAGATGGCGAAGGTCGACGATAGTTTCTTTCATCGGTTGGAAGAATCGTTCCTCGTAGAGGAGGACAAAAAGCACGAGCGTCACCCAATATTCGGGAATATTGTTGACGAGGTTGCCTACCATGAGAAATATCCTACAATATATCACCTCCGTAAGAAGCTTGTCGATTCAACTGATAAGGCTGATCTCAGACTCATCTATCTTGCCCTCGCACATATGATTAAGTTTCGTGGCCACTTCTTGATTGAAGGCGACCTCAACCCGGACAACTCAGATGTTGACAAGCTTTTTATACAGCTCGTCCAGACATATAACCAGCTGTTTGAAGAGAATCCCATCAATGCGAGTGGGGTTGATGCTAAGGCCATTTTGTCCGCCAGGTTGTCCAAATCTCGCAGACTGGAAAACCTGATCGCACAGCTTCCCGGTGAAAAGAAAAACGGGCTCTTCGGCAATCTCATCGCACTGTCCCTCGGCCTCACCCCAAACTTCAAGTCTAACTTCGACCTGGCCGAGGATGCGAAGCTCCAGCTGTCAAAAGATACATACGACGACGATTTGGACAATCTGCTTGCGCAAATAGGCGACCAGTATGCGGACCTGTTCCTGGCTGCCAAAAATCTGTCAGATGCAATCCTCCTGTCCGATATATTGCGTGTGAACACCGAAATCACGAAGGCACCGCTTAGCGCATCCATGATCAAGAGATACGACGAGCACCATCAGGACCTCACACTCCTCAAGGCGCTTGTTCGTCAGCAGCTTCCCGAGAAATATAAGGAAATTTTTTTCGATCAAAGCAAGAATGGATATGCTGGCTATATTGACGGTGGCGCTTCGCAGGAGGAGTTCTATAAATTCATTAAGCCGATTCTGGAGAAGATGGACGGAACGGAGGAGCTCCTCGTCAAGCTTAACCGGGAAGACCTGTTGCGGAAGCAGAGGACTTTTGATAACGGCTCTATTCCGCACCAAATCCATCTGGGTGAGTTGCACGCAATCTTGAGAAGACAAGAGGATTTCTACCCGTTCCTTAAGGATAACAGAGAGAAGATAGAAAAAATACTGACCTTCAGGATACCATACTATGTGGGCCCACTGGCGCGCGGAAATAGTCGTTTCGCATGGATGACTAGAAAGTCCGAAGAAACGATCACGCCATGGAATTTTGAGGAAGTGGTCGACAAGGGCGCCTCTGCCCAGAGCTTCATCGAAAGGATGACCAATTTTGACAAAAATCTGCCTAACGAAAAGGTGCTTCCGAAGCACAGCCTGTTGTATGAATACTTCACAGTTTATAACGAGCTCACTAAGGTCAAGTACGTCACGGAGGGCATGCGTAAGCCTGCTTTCCTGTCTGGTGAACAAAAAAAGGCGATTGTGGACCTCCTTTTCAAGACGAACCGTAAAGTTACTGTGAAGCAACTGAAAGAGGATTACTTTAAGAAAATTGAGTGCTTCGACAGTGTGGAGATTTCCGGTGTCGAGGACCGGTTTAACGCCAGCCTGGGTACGTATCATGACCTGCTTAAAATTATCAAGGATAAAGATTTCCTGGATAATGAAGAGAACGAAGATATACTGGAGGACATTGTGTTGACTTTGACCCTCTTCGAGGACAGAGAGATGATTGAGGAAAGACTGAAGACCTACGCACACCTTTTTGATGACAAGGTCATGAAACAACTCAAGCGCCGGCGCTATACTGGCTGGGGCCGGCTTTCTCGCAAGCTCATCAATGGGATTCGGGATAAGCAATCAGGCAAGACAATTTTGGACTTCCTCAAATCCGACGGATTCGCAAATAGGAATTTTATGCAGCTGATACATGACGACTCTTTGACATTCAAAGAAGACATACAGAAGGCTCAGGTCTCCGGCCAAGGAGATTCTTTGCACGAGCATATCGCTAACTTGGCAGGTAGCCCCGCCATAAAAAAGGGCATTCTTCAAACGGTAAAAGTTGTTGACGAACTCGTGAAGGTTATGGGCCGTCATAAGCCGGAAAACATTGTTATTGAAATGGCTAGGGAAAATCAGACGACCCAGAAGGGACAGAAAAATAGCAGGGAGCGGATGAAGAGAATTGAAGAGGGAATTAAGGAGCTTGGATCTCAGATTCTTAAGGAGCACCCTGTGGAGAACACCCAACTTCAGAATGAAAAGCTCTACCTTTACTACCTTCAAAACGGCCGGGATATGTACGTCGATCAGGAACTTGACATTAACCGGTTGAGCGATTATGACGTTGACCATATTGTGCCCCAATCTTTCCTTAAAGACGACTCTATCGACAATAAAGTGCTGACGCGCAGCGATAAAAATCGCGGTAAGTCGGATAATGTCCCGTCGGAAGAGGTGGTTAAAAAAATGAAGAACTATTGGAGGCAACTCCTGAATGCCAAGCTGATCACTCAGAGGAAATTCGACAATCTCACCAAGGCAGAAAGGGGTGGACTTAGCGAGCTCGACAAGGCCGGTTTTATCAAAAGACAGCTGGTGGAGACACGCCAAATCACCAAACACGTTGCCCAGATCCTGGATTCGAGGATGAACACGAAGTATGACGAGAACGACAAGTTGATTAGGGAAGTCAAGGTCATCACTTTGAAGTCCAAGCTGGTGAGCGACTTTCGCAAAGACTTCCAGTTTTACAAAGTCAGGGAAATTAATAACTACCACCACGCCCACGACGCCTACCTTAACGCCGTGGTTGGCACAGCACTCATCAAGAAATACCCTAAGCTCGAATCTGAGTTCGTCTATGGCGACTATAAGGTCTACGACGTTAGAAAAATGATCGCGAAATCTGAGCAGGAAATAGGCAAGGCAACTGCCAAGTACTTCTTCTATTCCAATATCATGAACTTTTTTAAGACGGAGATTACCCTGGCGAATGGTGAGATCCGCAAGCGCCCTTTGATTGAGACAAACGGAGAAACAGGAGAGATCGTATGGGACAAAGGGCGGGACTTTGCTACTGTTAGGAAGGTGCTCTCTATGCCACAAGTTAACATTGTCAAAAAAACTGAAGTGCAGACAGGTGGGTTTAGCAAGGAATCTATCCTGCCGAAGAGGAACTCTGACAAGCTGATCGCCCGCAAGAAAGATTGGGATCCGAAAAAGTACGGAGGATTCGACTCCCCCACAGTTGCGTACTCCGTGCTTGTCGTGGCCAAAGTGGAGAAGGGCAAGTCTAAGAAGCTCAAGAGCGTCAAAGAGTTGTTGGGGATCACGATTATGGAGCGGTCGTCTTTCGAAAAGAATCCGATAGATTTTCTCGAGGCCAAGGGTTATAAAGAAGTCAAGAAGGATCTTATCATCAAGCTCCCTAAGTACTCCCTCTTTGAGCTTGAAAACGGACGGAAAAGAATGCTGGCTTCAGCGGGTGAACTTCAGAAGGGTAATGAACTCGCTCTGCCCTCAAAATATGTGAATTTCCTTTACCTGGCATCACACTATGAGAAGCTTAAGGGGTCTCCAGAGGACAACGAGCAGAAGCAACTGTTCGTTGAACAACACAAGCACTACCTTGACGAGATTATCGAGCAAATCAGCGAGTTTAGCAAGCGCGTTATACTGGCAGACGCAAATCTTGATAAGGTCCTTAGCGCCTACAACAAGCATAGAGACAAACCCATCCGGGAGCAGGCCGAGAACATTATTCATCTCTTCACCTTGACGAATCTTGGGGCCCCGGCCGCGTTCAAGTACTTCGATACTACCATAGACAGAAAGCGCTATACATCGACAAAGGAAGTTCTTGACGCCACGCTGATCCACCAAAGTATAACAGGCCTCTATGAGACACGCATCGACCTTTCGCAGTTGGGCGGTGACCGCCCCAAAAAGAAGAGGAAAGTTGGCGGGTGAACTAGTGAGCTCGAATTTCCCCGATCGTTCAAACATTTGGCAATAAAGTTTCTTAAGATTGAATCCTGTTGCCGGTCTTGCGATGATTATCATATAATTTCTGTTGAATTACGTTAAGCATGTAATAATTAACATGTAATGCATGACGTTATTTATGAGATGGGTTTTTATGATTAGAGTCCCGCAATTATACATTTAATACGCGATAGAAAACAAAATATAGCGCGCAAACTAGGATAAATTATCGCGCGCGGTGTCATCTATGTTACTAGATCGGAAATTAATTCGTAATCATGGTCATAGCTGTTTCCTGTGTGAAATTGTTATCCGCTCACAATTCCACACAACATACGAGCCGGAAGCATAAAGTGTAAAGCCTGGGGTGCCTAATGAGTGAGCTAACTCACATTAATTGCGTTGCGCTCACTGCCCGCTTTCCAGTCGGGAAACCTGTCGTGCCAGCTGCATTAATGAATCGGCCAACGCGCGGGGAGAGGCGGTTTGCGTATTGGCTAGAGCAGCTTGCCAACATGGTGGAGCACGACACTCTCGTCTACTCCAAGAATATCAAAGATACAGTCTCAGAAGACCAAAGGGCTATTGAGACTTTTCAACAAAGGGTAATATCGGGAAACCTCCTCGGATTCCATTGCCCAGCTATCTGTCACTTCATCAAAAGGACAGTAGAAAAGGAAGGTGGCACCTACAAATGCCATCATTGCGATAAAGGAAAGGCTATCGTTCAAGATGCCTCTGCCGACAGTGGTCCCAAAGATGGACCCCCACCCACGAGGAGCATCGTGGAAAAAGAAGACGTTCCAACCACGTCTTCAAAGCAAGTGGATTGATGTGATAACATGGTGGAGCACGACACTCTCGTCTACTCCAAGAATATCAAAGATACAGTCTCAGAAGACCAAAGGGCTATTGAGACTTTTCAACAAAGGGTAATATCGGGAAACCTCCTCGGATTCCATTGCCCAGCTATCTGTCACTTCATCAAAAGGACAGTAGAAAAGGAAGGTGGCACCTACAAATGCCATCATTGCGATAAAGGAAAGGCTATCGTTCAAGATGCCTCTGCCGACAGTGGTCCCAAAGATGGACCCCCACCCACGAGGAGCATCGTGGAAAAAGAAGACGTTCCAACCACGTCTTCAAAGCAAGTGGATTGATGTGATATCTCCACTGACGTAAGGGATGACGCACAATCCCACTATCCTTCGCAAGACCTTCCTCTATATAAGGAAGTTCATTTCATTTGGAGAGGACACGCTGAAATCACCAGTCTCTCTCTACAAATCTATCTCTCTCGAGCTTTCGCAGATCCCGGGGGGCAATGAGATATGAAAAAGCCTGAACTCACCGCGACGTCTGTCGAGAAGTTTCTGATCGAAAAGTTCGACAGCGTCTCCGACCTGATGCAGCTCTCGGAGGGCGAAGAATCTCGTGCTTTCAGCTTCGATGTAGGAGGGCGTGGATATGTCCTGCGGGTAAATAGCTGCGCCGATGGTTTCTACAAAGATCGTTATGTTTATCGGCACTTTGCATCGGCCGCGCTCCCGATTCCGGAAGTGCTTGACATTGGGGAGTTTAGCGAGAGCCTGACCTATTGCATCTCCCGCCGTGCACAGGGTGTCACGTTGCAAGACCTGCCTGAAACCGAACTGCCCGCTGTTCTACAACCGGTCGCGGAGGCTATGGATGCGATCGCTGCGGCCGATCTTAGCCAGACGAGCGGGTTCGGCCCATTCGGACCGCAAGGAATCGGTCAATACACTACATGGCGTGATTTCATATGCGCGATTGCTGATCCCCATGTGTATCACTGGCAAACTGTGATGGACGACACCGTCAGTGCGTCCGTCGCGCAGGCTCTCGATGAGCTGATGCTTTGGGCCGAGGACTGCCCCGAAGTCCGGCACCTCGTGCACGCGGATTTCGGCTCCAACAATGTCCTGACGGACAATGGCCGCATAACAGCGGTCATTGACTGGAGCGAGGCGATGTTCGGGGATTCCCAATACGAGGTCGCCAACATCTTCTTCTGGAGGCCGTGGTTGGCTTGTATGGAGCAGCAGACGCGCTACTTCGAGCGGAGGCATCCGGAGCTTGCAGGATCGCCACGACTCCGGGCGTATATGCTCCGCATTGGTCTTGACCAACTCTATCAGAGCTTGGTTGACGGCAATTTCGATGATGCAGCTTGGGCGCAGGGTCGATGCGACGCAATCGTCCGATCCGGAGCCGGGACTGTCGGGCGTACACAAATCGCCCGCAGAAGCGCGGCCGTCTGGACCGATGGCTGTGTAGAAGTACTCGCCGATAGTGGAAACCGACGCCCCAGCACTCGTCCGAGGGCAAAGAAATAGAGTAGATGCCGACCGGATCTGTCGATCGACAAGCTCGAGTTTCTCCATAATAATGTGTGAGTAGTTCCCAGATAAGGGAATTAGGGTTCCTATAGGGTTTCGCTCATGTGTTGAGCATATAAGAAACCCTTAGTATGTATTTGTATTTGTAAAATACTTCTATCAATAAAATTTCTAATTCCTAAAACCAAAATCCAGTACTAAAATCCAGATCCCCCGAATTAATTCGGCGTTAATTCAGTACATTAAAAACGTCCGCAATGTGTTATTAAGTTGTCTAAGCGTCAATTTGTTTACACCACAATATATCCTGCCACCAGCCAGCCAACAGCTCCCCGACCGGCAGCTCGGCACAAAATCACCACTCGATACAGGCAGCCCATCAGTCCGGGACGGCGTCAGCGGGAGAGCCGTTGTAAGGCGGCAGACTTTGCTCATGTTACCGATGCTATTCGGAAGAACGGCAACTAAGCTGCCGGGTTTGAAACACGGATGATCTCGCGGAGGGTAGCATGTTGATTGTAACGATGACAGAGCGTTGCTGCCTGTGATCACCGCGGTTTCAAAATCGGCTCCGTCGATACTATGTTATACGCCAACTTTGAAAACAACTTTGAAAAAGCTGTTTTCTGGTATTTAAGGTTTTAGAATGCAAGGAACAGTGAATTGGAGTTCGTCTTGTTATAATTAGCTTCTTGGGGTATCTTTAAATACTGTAGAAAAGAGGAAGGAAATAATAAATGGCTAAAATGAGAATATCACCGGAATTGAAAAAACTGATCGAAAAATACCGCTGCGTAAAAGATACGGAAGGAATGTCTCCTGCTAAGGTATATAAGCTGGTGGGAGAAAATGAAAACCTATATTTAAAAATGACGGACAGCCGGTATAAAGGGACCACCTATGATGTGGAACGGGAAAAGGACATGATGCTATGGCTGGAAGGAAAGCTGCCTGTTCCAAAGGTCCTGCACTTTGAACGGCATGATGGCTGGAGCAATCTGCTCATGAGTGAGGCCGATGGCGTCCTTTGCTCGGAAGAGTATGAAGATGAACAAAGCCCTGAAAAGATTATCGAGCTGTATGCGGAGTGCATCAGGCTCTTTCACTCCATCGACATATCGGATTGTCCCTATACGAATAGCTTAGACAGCCGCTTAGCCGAATTGGATTACTTACTGAATAACGATCTGGCCGATGTGGATTGCGAAAACTGGGAAGAAGACACTCCATTTAAAGATCCGCGCGAGCTGTATGATTTTTTAAAGACGGAAAAGCCCGAAGAGGAACTTGTCTTTTCCCACGGCGACCTGGGAGACAGCAACATCTTTGTGAAAGATGGCAAAGTAAGTGGCTTTATTGATCTTGGGAGAAGCGGCAGGGCGGACAAGTGGTATGACATTGCCTTCTGCGTCCGGTCGATCAGGGAGGATATCGGGGAAGAACAGTATGTCGAGCTATTTTTTGACTTACTGGGGATCAAGCCTGATTGGGAGAAAATAAAATATTATATTTTACTGGATGAATTGTTTTAGTACCTAGAATGCATGACCAAAATCCCTTAACGCTGAGAGATCCCCTCATAATTTCCCCAAAGCGTAACCATGTGTGAATAAATTTTGAGCTAGTAGGGTTGCAGCCACGAGTAAGTCTTCCCTTGTTATTGTGTAGCCAGAATGCCGCAAAACTTCCATGCCTAAGCGAACTGTTGAGAGTACGTTTCGATTTCTGACTGTGTTAGCCTGGAAGTGCTTGTCCCAACCTTGTTTCTGAGCATGAACGCCCGCAAGCCAACATGTTAGTTGAAGCATCAGGGCGATTAGCAGCATGATATCAAAACGCTCTGAGCTGCTCGTTCGGCTATGGCGTAGGCCTAGTCCGTAGGCAGGACTTTTCAAGTCTCGGAAGGTTTCTTCAATCTGCATTCGCTTCGAATAGATATTAACAAGTTGTTTGGGTGTTCGAATTTCAACAGGTAAGTTAGTTGCTAGAATCCATGGCTCCTTTGCCGACGCTGAGTAGATTTTAGGTGACGGGTGGTGACAATGAGTCCGTGTCGAGCGCTGATTTTTTCGGCCTTTAGAGCGAGATTTATACAATAGAATTTGGCATGAGATTGGATTGCTTTTAGTCAGCCTCTTATAGCCTAAAGTCTTTGAGTGACTAGATGACATATCATGTAAGTTGCTGATAGGTTTCCAGTTTTCCGCTCCTAGGTCTGCATATTGTACTTTTCCTCTTACTCGACTTAACCAGTACCAACCCAGCTTCTCAACGGATTTATACCATGGCACTTTAAAGCCAGCATCACTGACAATGAGCGGTGTGGTGTTACTCGGTAGAATGCTCGCAAGGTCGGCTAGAAATTGGTCATGAGCTTTCTTTGAACATTGCTCTGAAAGCGGGAACGCTTTCTCATAAAGAGTAACAGAACGACCGTGTAGTGCGACTGAAGCTCGCAATACCATAAGCCGTTTTTGCTCACGGATATCAGACCAGTCAACAAGTACAATGGGCATCGTATTGCCCGAACAGATAAAGCTAGCATGCCAACGGTATACAGCGAGTCGCTCTTTGTGGAGGTGACGATTACCTAACAATCGGTCGATTCGTTTGATGTTATGTTTTGTTCTCGCTTTGGTTGGCAGGTTACGGCCAAGTTCGGTAAGAGTGAGAGTTTTACAGTCAAGTAAGGCGTGGCAAGCCAACGTTAAGCTGTTGAGTCGTTTTAAGTGTAATTCGGGGCAGAATTGGTAAAGAGAGTCGTGTAAAATATCGAGTTCGCACATTTTGTTGTCTGATTATTGATTTTTGGCGAAACCATTTGATCATATGACAAGATGTGTATCTACCTTAACTTAATGATTTTGATAAAAATCATTAGGGGATTCATCAGCCCTTAACGTGAGTTTTCGTTCCACTGAGCGTCAGACCCCGTAGAAAAGATCAAAGGATCTTCTTGAGATCCTTTTTTTCTGCGCGTAATCTGCTGCTTGCAAACAAAAAAACCACCGCTACCAGCGGTGGTTTGTTTGCCGGATCAAGAGCTACCAACTCTTTTTCCGAAGGTAACTGGCTTCAGCAGAGCGCAGATACCAAATACTGTCCTTCTAGTGTAGCCGTAGTTAGGCCACCACTTCAAGAACTCTGTAGCACCGCCTACATACCTCGCTCTGCTAATCCTGTTACCAGTGGCTGCTGCCAGTGGCGATAAGTCGTGTCTTACCGGGTTGGACTCAAGACGATAGTTACCGGATAAGGCGCAGCGGTCGGGCTGAACGGGGGGTTCGTGCACACAGCCCAGCTTGGAGCGAACGACCTACACCGAACTGAGATACCTACAGCGTGAGCTATGAGAAAGCGCCACGCTTCCCGAAGGGAGAAAGGCGGACAGGTATCCGGTAAGCGGCAGGGTCGGAACAGGAGAGCGCACGAGGGAGCTTCCAGGGGGAAACGCCTGGTATCTTTATAGTCCTGTCGGGTTTCGCCACCTCTGACTTGAGCGTCGATTTTTGTGATGCTCGTCAGGGGGGCGGAGCCTATGGAAAAACGCCAGCAACGCGGCCTTTTTACGGTTCCTGGCCTTTTGCTGGCCTTTTGCTCACATGTTCTTTCCTGCGTTATCCCCTGATTCTGTGGATAACCGTATTACCGCCTTTGAGTGAGCTGATACCGCTCGCCGCAGCCGAACGACCGAGCGCAGCGAGTCAGTGAGCGAGGAAGCGGAAGAGCGCCTGATGCGGTATTTTCTCCTTACGCATCTGTGCGGTATTTCACACCGCATATGGTGCACTCTCAGTACAATCTGCTCTGATGCCGCATAGTTAAGCCAGTATACACTCCGCTATCGCTACGTGACTGGGTCATGGCTGCGCCCCGACACCCGCCAACACCCGCTGACGCGCCCTGACGGGCTTGTCTGCTCCCGGCATCCGCTTACAGACAAGCTGTGACCGTCTCCGGGAGCTGCATGTGTCAGAGGTTTTCACCGTCATCACCGAAACGCGCGAGGCAGGGTGCCTTGATGTGGGCGCCGGCGGTCGAGTGGCGACGGCGCGGCTTGTCCGCGCCCTGGTAGATTGCCTGGCCGTAGGCCAGCCATTTTTGAGCGGCCAGCGGCCGCGATAGGCCGACGCGAAGCGGCGGGGCGTAGGGAGCGCAGCGACCGAAGGGTAGGCGCTTTTTGCAGCTCTTCGGCTGTGCGCTGGCCAGACAGTTATGCACAGGCCAGGCGGGTTTTAAGAGTTTTAATAAGTTTTAAAGAGTTTTAGGCGGAAAAATCGCCTTTTTTCTCTTTTATATCAGTCACTTACATGTGTGACCGGTTCCCAATGTACGGCTTTGGGTTCCCAATGTACGGGTTCCGGTTCCCAATGTACGGCTTTGGGTTCCCAATGTACGTGCTATCCACAGGAAAGAGACCTTTTCGACCTTTTTCCCCTGCTAGGGCAATTTGCCCTAGCATCTGCTCCGTACATTAGGAACCGGCGGATGCTTCGCCCTCGATCAGGTTGCGGTAGCGCATGACTAGGATCGGGCCAGCCTGCCCCGCCTCCTCCTTCAAATCGTACTCCGGCAGGTCATTTGACCCGATCAGCTTGCGCACGGTGAAACAGAACTTCTTGAACTCTCCGGCGCTGCCACTGCGTTCGTAGATCGTCTTGAACAACCATCTGGCTTCTGCCTTGCCTGCGGCGCGGCGTGCCAGGCGGTAGAGAAAACGGCCGATGCCGGGATCGATCAAAAAGTAATCGGGGTGAACCGTCAGCACGTCCGGGTTCTTGCCTTCTGTGATCTCGCGGTACATCCAATCAGCTAGCTCGATCTCGATGTACTCCGGCCGCCCGGTTTCGCTCTTTACGATCTTGTAGCGGCTAATCAAGGCTTCACCCTCGGATACCGTCACCAGGCGGCCGTTCTTGGCCTTCTTCGTACGCTGCATGGCAACGTGCGTGGTGTTTAACCGAATGCAGGTTTCTACCAGGTCGTCTTTCTGCTTTCCGCCATCGGCTCGCCGGCAGAACTTGAGTACGTCCGCAACGTGTGGACGGAACACGCGGCCGGGCTTGTCTCCCTTCCCTTCCCGGTATCGGTTCATGGATTCGGTTAGATGGGAAACCGCCATCAGTACCAGGTCGTAATCCCACACACTGGCCATGCCGGCCGGCCCTGCGGAAACCTCTACGTGCCCGTCTGGAAGCTCGTAGCGGATCACCTCGCCAGCTCGTCGGTCACGCTTCGACAGACGGAAAACGGCCACGTCCATGATGCTGCGACTATCGCGGGTGCCCACGTCATAGAGCATCGGAACGAAAAAATCTGGTTGCTCGTCGCCCTTGGGCGGCTTCCTAATCGACGGCGCACCGGCTGCCGGCGGTTGCCGGGATTCTTTGCGGATTCGATCAGCGGCCGCTTGCCACGATTCACCGGGGCGTGCTTCTGCCTCGATGCGTTGCCGCTGGGCGGCCTGCGCGGCCTTCAACTTCTCCACCAGGTCATCACCCAGCGCCGCGCCGATTTGTACCGGGCCGGATGGTTTGCGACCGTCACGCCGATTCCTCGGGCTTGGGGGTTCCAGTGCCATTGCAGGGCCGGCAGACAACCCAGCCGCTTACGCCTGGCCAACCGCCCGTTCCTCCACACATGGGGCATTCCACGGCGTCGGTGCCTGGTTGTTCTTGATTTTCCATGCCGCCTCCTTTAGCCGCTAAAATTCATCTACTCATTTATTCATTTGCTCATTTACTCTGGTAGCTGCGCGATGTATTCAGATAGCAGCTCGGTAATGGTCTTGCCTTGGCGTACCGCGTACATCTTCAGCTTGGTGTGATCCTCCGCCGGCAACTGAAAGTTGACCCGCTTCATGGCTGGCGTGTCTGCCAGGCTGGCCAACGTTGCAGCCTTGCTGCTGCGTGCGCTCGGACGGCCGGCACTTAGCGTGTTTGTGCTTTTGCTCATTTTCTCTTTACCTCATTAACTCAAATGAGTTTTGATTTAATTTCAGCGGCCAGCGCCTGGACCTCGCGGGCAGCGTCGCCCTCGGGTTCTGATTCAAGAACGGTTGTGCCGGCGGCGGCAGTGCCTGGGTAGCTCACGCGCTGCGTGATACGGGACTCAAGAATGGGCAGCTCGTACCCGGCCAGCGCCTCGGCAACCTCACCGCCGATGCGCGTGCCTTTGATCGCCCGCGACACGACAAAGGCCGCTTGTAGCCTTCCATCCGTGACCTCAATGCGCTGCTTAACCAGCTCCACCAGGTCGGCGGTGGCCCATATGTCGTAAGGGCTTGGCTGCACCGGAATCAGCACGAAGTCGGCTGCCTTGATCGCGGACACAGCCAAGTCCGCCGCCTGGGGCGCTCCGTCGATCACTACGAAGTCGCGCCGGCCGATGGCCTTCACGTCGCGGTCAATCGTCGGGCGGTCGATGCCGACAACGGTTAGCGGTTGATCTTCCCGCACGGCCGCCCAATCGCGGGCACTGCCCTGGGGATCGGAATCGACTAACAGAACATCGGCCCCGGCGAGTTGCAGGGCGCGGGCTAGATGGGTTGCGATGGTCGTCTTGCCTGACCCGCCTTTCTGGTTAAGTACAGCGATAACCTTCATGCGTTCCCCTTGCGTATTTGTTTATTTACTCATCGCATCATATACGCAGCGACCGCATGACGCAAGCTGTTTTACTCAAATACACATCACCTTTTTAGACGGCGGCGCTCGGTTTCTTCAGCGGCCAAGCTGGCCGGCCAGGCCGCCAGCTTGGCATCAGACAAACCGGCCAGGATTTCATGCAGCCGCACGGTTGAGACGTGCGCGGGCGGCTCGAACACGTACCCGGCCGCGATCATCTCCGCCTCGATCTCTTCGGTAATGAAAAACGGTTCGTCCTGGCCGTCCTGGTGCGGTTTCATGCTTGTTCCTCTTGGCGTTCATTCTCGGCGGCCGCCAGGGCGTCGGCCTCGGTCAATGCGTCCTCACGGAAGGCACCGCGCCGCCTGGCCTCGGTGGGCGTCACTTCCTCGCTGCGCTCAAGTGCGCGGTACAGGGTCGAGCGATGCACGCCAAGCAGTGCAGCCGCCTCTTTCACGGTGCGGCCTTCCTGGTCGATCAGCTCGCGGGCGTGCGCGATCTGTGCCGGGGTGAGGGTAGGGCGGGGGCCAAACTTCACGCCTCGGGCCTTGGCGGCCTCGCGCCCGCTCCGGGTGCGGTCGATGATTAGGGAACGCTCGAACTCGGCAATGCCGGCGAACACGGTCAACACCATGCGGCCGGCCGGCGTGGTGGTGTCGGCCCACGGCTCTGCCAGGCTACGCAGGCCCGCGCCGGCCTCCTGGATGCGCTCGGCAATGTCCAGTAGGTCGCGGGTGCTGCGGGCCAGGCGGTCTAGCCTGGTCACTGTCACAACGTCGCCAGGGCGTAGGTGGTCAAGCATCCTGGCCAGCTCCGGGCGGTCGCGCCTGGTGCCGGTGATCTTCTCGGAAAACAGCTTGGTGCAGCCGGCCGCGTGCAGTTCGGCCCGTTGGTTGGTCAAGTCCTGGTCGTCGGTGCTGACGCGGGCATAGCCCAGCAGGCCAGCGGCGGCGCTCTTGTTCATGGCGTAATGTCTCCGGTTCTAGTCGCAAGTATTCTACTTTATGCGACTAAAACACGCGACAAGAAAACGCCAGGAAAAGGGCAGGGCGGCAGCCTGTCGCGTAACTTAGGACTTGTGCGACATGTCGTTTTCAGAAGACGGCTGCACTGAACGTCAGAAGCCGACTGCACTATAGCAGCGGAGGGGTTGGATCAAAGTACTTTGATCCCGAGGGGAACCCTGTGGTTGGCATGCACATACAAATGGACGAACGGATAAACCTTTTCACGCCCTTTTAAATATCCGTTATTCTAATAAACGCTCTTTTCTCTTA
