# Supplementary material for: Methylviologen resistance in loss-of-function mutants of the polyamine transporter gene OsLAT5
Source: PLoS One. 2026 Apr 16;21(4):e0346828. doi: 10.1371/journal.pone.0346828 (PMC13086316; doi:10.1371/journal.pone.0346828)
Supplement: S5 File — Alignments are made using MUSCLE (Multiple Sequence Comparison by Log- Expectation) algorithm. Non-mutated nucleotides that are shaded in BLACK using pyBoxshade program. Deleted nucleotides in mutant alleles are represented with the “–“ symbol. Inserted nucleotides in mutant alleles are non-shaded. Two guide RNAs were used for knock out of each rice gene. Selected guide RNAs are colored in red, while PAM sites are colored in green. (DOCX) [file pone.0346828.s005.docx]

**Supporting Information S5. Alignments of coding sequences (CDS) of rice *LAT1*, *LAT5*, *LAT7* wild-type and the mutant alleles used in this study.** Alignments are made using MUSCLE (Multiple Sequence Comparison by Log- Expectation) algorithm. Non-mutated nucleotides that are shaded in BLACK using pyBoxshade program. Deleted nucleotides in mutant alleles are represented with the “**–“** symbol. Inserted nucleotides in mutant alleles are non-shaded. Two guide RNAs were used for knock out of each rice gene. Selected guide RNAs are colored in **red**, while PAM sites are colored in **green**.

***LAT1*/*PUT1* (Os02g0700500; LOC_Os02g47210)**

....:...10....:...20....:...30....:...40....:...50....:...60
*LAT1* **ATGGCGGACACCGGCGGACGGCCGGAGGTGTCGCTGGCCACGGTCCGGTCGCCGGGCCAC***lat1-1* **ATGGCGGACACCGGCGGACGGCCGGAGGTGTCGCTGGCCACGGTCCGGTCGCCGGGCCAC***lat1-2* **ATGGCGGACACCGGCGGACGGCCGGAGGTGTCGCTGGCCACGGTCCGGTCGCCGGGCCAC** ....:...70....:...80....:...90....:..100....:..110....:..120
*LAT1* **CCGGCAGCTTCTACGACGGCAGCGGCGGCGGCGGATCTCGGCCACGCTGACACCGGGCAA***lat1-1* **CCGGCAGCTTCTACGACGGCAGCGGCGGCGGCGGATCTCGGCCACGCTGACACCGGGCAA***lat1-2* **CCGGCAGCTTCTACGACGGCAGCGGCGGCGGCGGATCTCGGCCACGCTGACACCGGGCAA** ....:..130....:..140....:..150....:..160....:..170....:..180
*LAT1* **GAGAAGCCCACCGTCGAGAGCGCCCAACCGGCGAACGGTGCCGCTCCGATGGGCGAGTGC***lat1-1* **GAGAAGCCCACCGTCGAGAGCGCCCAACCGGCGAACGGTGCCGCTCCGATGGGCGAGTGC***lat1-2* **GAGAAGCCCACCGTCGAGAGCGCCCAACCGGCGAACGGTGCCGCTCCGATGGGCGAGTGC** ....:..190....:..200....:..210....:..220....:..230....:..240
*LAT1* **GGCACGGAGTACAGGGGCCTCCCCGACGGCGACGCCGGCGGGCCAATGCCGTCGTCGGCA***lat1-1* **GGCACGGAGTACAGGGGCCTCCCCGACGGCGACGCCGGCGGGCCAATGCCGTCGTCGGCA***lat1-2* **GGCACGGAGTACAGGGGCCTCCCCGACGGCGACGCCGGCGGGCCAATGCCGTCGTCGGCA** ....:..250....:..260....:..270....:..280....:..290....:..300
*LAT1* **CGCACGGTTTCGATGATCCCGCTCATCTTCCTCATCTTCTACGAGGTGTCCGGCGGGCCG***lat1-1* **CGCACGGTTTCGATGATCCCGCTCATCTTCCTCATCTTCTACGAGGTGTCCGGCGGGCCG***lat1-2* **CGCACGGTTTCGATGATCCCGCTCATCTTCCTCATCTTCTACGAGGTGTCCGGCGGGCCG** ....:..310....:..320....:..330....:..340....:..350....:..360
*LAT1* **TTCGGGATCGAGGACAGCGTGGGCGCGGCCGGGCCGCTGCTCGCCATCATCGGCTTCTTG***lat1-1* **TTCGGGATCGAGGACAGCGTGGGCGCGGCCGGGCCGCTGCTCGCCATCATCGGCTTCTTG***lat1-2* **TTCGGGATCGAGGACAGCGTGGGCGCGGCCGGGCCGCTGCTCGCCATCATCGGCTTCTTG** ....:..370....:..380....:..390....:..400....:..410....:..420
*LAT1* **GTCCTCCCCGTCATCTGGAGCATCCCGGAGGCGCTGATCACGGCGGAGCTGGGCGCCATG***lat1-1* **GTCCTCCCCGTCATCTGGAGCATCCCGGAGGCGCTGATCACGGCGGAGCTGGGCGCCATG***lat1-2* **GTCCTCCCCGTCATCTGGAGCATCCCGGAGGCGCTGATCACGGCGGAGCTGGGCGCCATG** ....:..430....:..440....:..450....:..460....:..470....:..480
*LAT1*  **TTCCCGGAGAACGGCGGGTACGTCGTGTGGGTGGCGTCGGCGCTCGGCCCGTACTGGGGG***lat1-1* **TTCCCGGAGAACGGCGGGTACGTCGTGTGGGTGGCGTCGGCGCTCGGCCCGTACTGGGGG***lat1-2* **TTCCCGGAGAACGGCGGGTACGTCGTGTGGGTGGCGTCGGCGCTCGGCCCGTACTGGGGG** ....:..490....:..500....:..510....:..520....:..530....:..540
*LAT1*  **TTCCAGCAAGGGTGGATGAAGTGGTTGAGCGGCGTCATCGACAACGCGCTCTACCCCGTC***lat1-1* **TTCCAGCAAGGGTGGATGAAGTGGTTGAGCGGCGTCATCGACAACGCGCTCTACCCCGTC***lat1-2* **TTCCAGCAAGGGTGGATGAAGTGGTTGAGCGGCGTCATCGACAACGCGCTCTACCCCGTC**

....:..550....:..560....:..570....:..580....:..590....:..600
*LAT1*  **CTCTTCTTGGACTACCTCAAGTCCGGCGTCCCGGCGCTCGGCGGAGGCGCGCCGAGGGCG***lat1-1* **CTCTTCTTGGACTACCTCAAGTCCGGCGTCCCGGCGCTCGGCGGAGGCGCGCCGAGGGCG***lat1-2* **CTCTTCTTGGACTACCTCAAGTCCGGCGTCCCGGCGCTCGGCGGAGGCGCGCCGAGGGCG** ....:..610....:..620....:..630....:..640....:..650....:..660
*LAT1*  **TTCGCCGTCGTCGGCCTGACGGCCGTGCTGACATTGCTGAATTACCGGGGGCTCACCGTC***lat1-1* **TTCGCCGTCGTCGGCCTGACGGCCGTGCTGACATTGCTGAATTACCGGGGGCTCACCGTC***lat1-2* **TTCGCCGTCGTCGGCCTGACGGCCGTGCTGACATTGCTGAATTACCGGGGGCTCACCGTC** ....:..670....:..680....:..690....:..700....:..710....:..720
*LAT1*  **GTCGGATGGGTGGCGATCTGCCTTGGCGTCTTCTCCCTCCTCCCTTTTTTCGTCATGGGG***lat1-1* **GTCGGATGGGTGGCGATCTGCCTTGGCGTCTTCTCCCTCCTCCCTTTTTTCGTCATGGGG***lat1-2* **GTCGGATGGGTGGCGATCTGCCTTGGCGTCTTCTCCCTCCTCCCTTTTTTCGTCATGGGG**

....:..730....:..740....:..750....:..760....:..770....:..780
*LAT1*  **CTCATCGCGCTCCCCAAGCTCCGGCCGGCGAGGTGGCTCGTGATCGACCTCCACAACGTC***lat1-1* **CTCATCGCGCTCCCCAAGCTCCGGCCGGCGAGGTGGCTCGTGATCGACCTCCACAACGTC***lat1-2* **CTCATCGCGCTCCCCAAGCTCCGGCCGGCGAGGTGGCTCGTGATCGACCTCCACAACGTC** ....:..790....:..800....:..810....:..820....:..830....:..840
*LAT1*  **GATTGGAATCTGTACCTGAACACTCTGTTCTGGAACCTCAACTACTGGGATTCGATCAGC***lat1-1* **GATTGGAATCTGTACCTGAACACTCTGTTCTGGAACCTCAACTACTGGGATTCGATCAGC***lat1-2* **GATTGGAATCTGTACCTGAACACTCTGTTCTGGAACCTCAACTACTGGGATTCGATCAGC** ....:..850....:..860....:..870....:..880....:..890....:..900
*LAT1*  **ACGTTGGCCGGCGAGGTGAAGAATCCCGGCAAGACGCTGCCCAAGGCGCTGTTCTACGCG***lat1-1* **ACGTTGGCCGGCGAGGTGAAGAATCCCGGCAAGACGCTGCCCAAGGCGCTGTTCTACGCG***lat1-2* **ACGTTGGCCGGCGAGGTGAAGAATCCCGGCAAGACGCTGCCCAAGGCGCTGTTCTACGCG** ....:..910....:..920....:..930....:..940....:..950....:..960
*LAT1*  **GTCATCTTCGTGGTGGTCGCCTACCTGTACCCTCTCCTCGCCGGGACGGGAGCCGTGCCG***lat1-1* **GTCATCTTCGTGGTGGTCGCCTACCTGTACCCTCTCCTCGCCGGGACGGGAGCCGTGCCG***lat1-2* **GTCATCTTCGTGGTGGTCGCCTACCTGTACCCTCTCCTCGCCGGGACGGGAGCCGTGCCG** ....:..970....:..980....:..990....:.1000....:.1010....:.1020
*LAT1*  **CTGGACAGGGGGCAGTGGACAGACGGCTACTTCGCGGACATCGCGAAGCTGCTCGGCGGC***lat1-1* **CTGGACAGGGGGCAGTGGACAGACGGCTACTTCGCGGACATCGCGAAGCTGCTCGGCGGC***lat1-2* **CTGGACAGGGGGCAGTGGACAGACGGCTACTTCGCGGACATCGCGAAGCTGCTCGGCGGC** ....:.1030....:.1040....:.1050....:.1060....:.1070....:.1080
*LAT1*  **GCGTGGCTGATGTGGTGGGTGCAGTCGGCGGCGGCGCTGTCGAACATGGGCATGTTCGTG***lat1-1* **GCGTGGCTGATGTGGTGGGTGCAGTCGGCGGCGGCGCTGTCGAACATGGGCATGTTCGTG***lat1-2* **GCGTGGCTGATGTGGTGGGTGCAGTCGGCGGCGGCGCTGTCGAACATGGGCATGTTCGTG** ....:.1090....:.1100....:.1110....:.1120....:.1130....:.1140
*LAT1*  **GCGGAGATGAGCAGCGACTCGTACCAGC-TGCTGGGCATGGCGGAGCGGGGCATGCTCCC***lat1-1* **GCGGAGATGAGCAGCGACTCGTA------TGCTGGGCATGGCGGAGCGGGGCATGCTCCC***lat1-2* **GCGGAGATGAGCAGCGACTCGTACCAGCTTGCTGGGCATGGCGGAGCGGGGCATGCTCCC** ....:.1150....:.1160....:.1170....:.1180....:.1190....:.1200
*LAT1*  **GTCCTTCTTCGCGGCGCGGTCGCGGTACGGCACGCCGCTGGCGGGCATCCTCTTCTCGGC***lat1-1* **GTCCTTCTTCGCGGCGCGGTCGCGGTACGGCACGCCGCTGGCGGGCATCCTCTTCTCGGC***lat1-2* **GTCCTTCTTCGCGGCGCGGTCGCGGTACGGCACGCCGCTGGCGGGCATCCTCTTCTCGGC** ....:.1210....:.1220....:.1230....:.1240....:.1250....:.1260
*LAT1*  **CTCCGGCGTGCTGCTGCTCTCGATGATGAGCTTCCAGGAGATCGTGGCGGCCGAGAACTT***lat1-1* **CTCCGGCGTGCTGCTGCTCTCGATGATGAGCTTCCAGGAGATCGTGGCGGCCGAGAACTT***lat1-2* **CTCCGGCGTGCTGCTGCTCTCGATGATGAGCTTCCAGGAGATCGTGGCGGCCGAGAACTT** ....:.1270....:.1280....:.1290....:.1300....:.1310....:.1320
*LAT1*  **CCTCTACTGCTTCGGCATGCTCCTCGAGTTCGTCGCCTTCATCCTGCACCGGGTGAGGCG***lat1-1* **CCTCTACTGCTTCGGCATGCTCCTCGAGTTCGTCGCCTTCATCCTGCACCGGGTGAGGCG***lat1-2* **CCTCTACTGCTTCGGCATGCTCCTCGAGTTCGTCGCCTTCATCCTGCACCGGGTGAGGCG** ....:.1330....:.1340....:.1350....:.1360....:.1370....:.1380
*LAT1*  **CCCCGACGCGGCGCGCCCATACAGGGTGCCGCTGGGCACAGCCGGGTGCGTGGCGATGCT***lat1-1* **CCCCGACGCGGCGCGCCCATACAGGGTGCCGCTGGGCACAGCCGGGTGCGTGGCGATGCT***lat1-2* **CCCCGACGCGGCGCGCCCATACAGGGTGCCGCTGGGCACAGCCGGGTGCGTGGCGATGCT** ....:.1390....:.1400....:.1410....:.1420....:.1430....:.1440
*LAT1*  **GGTGCCGCCGACGGCGCTGATCGCCGTGGTGCTCGCGCTGTCCACGCTGAAGGTGGCGGT***lat1-1* **GGTGCCGCCGACGGCGCTGATCGCCGTGGTGCTCGCGCTGTCCACGCTGAAGGTGGCGGT***lat1-2* **GGTGCCGCCGACGGCGCTGATCGCCGTGGTGCTCGCGCTGTCCACGCTGAAGGTGGCGGT**

....:.1450....:.1460....:.1470....:.1480....:.1490....:.1500
*LAT1*  **GGTGAGCCTCGGCGCGGTGGCCATGGGGCTCGTGCTGCAGCCGGCGCTGAGGTTCGTGGA***lat1-1* **GGTGAGCCTCGGCGCGGTGGCCATGGGGCTCGTGCTGCAGCCGGCGCTGAGGTTCGTGGA***lat1-2* **GGTGAGCCTCGGCGCGGTGGCCATGGGGCTCGTGCTGCAGCCGGCGCTGAGGTTCGTGGA** ....:.1510....:.1520....:.1530....:.1540....:.1550....:.1560
*LAT1*  **GAAGAAGCGGTGGCTGAGGTTCTCCGTTAACCCGGATCTCCCGGAGATCGGCGTGATTCG***lat1-1* **GAAGAAGCGGTGGCTGAGGTTCTCCGTTAACCCGGATCTCCCGGAGATCGGCGTGATTCG***lat1-2* **GAAGAAGCGGTGGCTGAGGTTCTCCGTTAACCCGGATCTCCCGGAGATCGGCGTGATTCG** ....:.1570....:.1580....:.1590....:..
*LAT1*  **CCCGCCCGCCGCGCCGGACGAGCCGTTGGTTCCGTAG***lat1-1* **CCCGCCCGCCGCGCCGGACGAGCCGTTGGTTCCGTAG***lat1-2* **CCCGCCCGCCGCGCCGGACGAGCCGTTGGTTCCGTAG**

***LAT5*/*PUT3*/*PAR1* (Os03g0576900; LOC_Os03g37984)**

....:...10....:...20....:...30....:...40....:...50....:...60
*LAT5*  **ATGACGAACGCATGGATCTCGCCGTCGGTGGTCGCCCTCTGCCCCTCTCCCCTCCCCTCA***lat5-1* **ATGACGAACGCATGGATCTCGCCGTCGGTGGTCGCCCTCTGCCCCTCTCCCCTCCCCTCA***lat5-2* **ATGACGAACGCATGGATCTCGCCGTCGGTGGTCGCCCTCTGCCCCTCTCCCCTCCCCTCA** ....:...70....:...80....:...90....:..100....:..110....:..120
*LAT5*  **TCTCGCCTCCCGGGTTCTGTCCTCTCCTGCTGGCCGGACAGCCGCGGCATCCGGCGCGGG***lat5-1* **TCTCGCCTCCCGGGTTCTGTCCTCTCCTGCTGGCCGGACAGCCGCGGCATCCGGCGCGGG***lat5-2* **TCTCGCCTCCCGGGTTCTGTCCTCTCCTGCTGGCCGGACAGCCGCGGCATCCGGCGCGGG** ....:..130....:..140....:..150....:..160....:..170....:..180
*LAT5*  **GCTGGCGAGGGGACAGCCGGACAGACGCTGCGGCCGGCGAGGGGATTCACTGTAGAGAAA***lat5-1* **GCTGGCGAGGGGACAGCCGGACAGACGCTGCGGCCGGCGAGGGGATTCACTGTAGAGAAA***lat5-2* **GCTGGCGAGGGGACAGCCGGACAGACGCTGCGGCCGGCGAGGGGATTCACTGTAGAGAAA** ....:..190....:..200....:..210....:..220....:..230....:..240
*LAT5*  **TTAAGGAACACAGCAATAACACGAGCTAACTCAGCCTGTCTTCCAATGGAGGATTGTGTT**lat5-1 **TTAAGGAACACAGCAATAACACGAGCTAACTCAGCCTGTCTTCCAATGGAGGATTGTGTT***lat5-2* **TTAAGGAACACAGCAATAACACGAGCTAACTCAGCCTGTCTTCCAATGGAGGATTGTGTT** ....:..250....:..260....:..270....:..280....:..290....:..300
*LAT5*  **GGTATCAAGTACAGCAGTGTCAATGAGGGCGAAGAGCGTAAGGGAGGCCATGGCGTCCCA***lat5-1* **GGTATCAAGTACAGCAGTGTCAATGAGGGCGAAGAGCGTAAGGGA-GCCATGGCGTCCCA***lat5-2* **GGTATCAAGTACAGCAGTGTCAATGAGGGCGAAGAGCGTAAGGGA-------GCGTCCCA** ....:..310....:..320....:..330....:..340....:..350....:..360
*LAT5*  **AAGGTTTCCATCATCCCACTCATTTTCCTCATATTCTATGAAGTTTCTGGGGGTCCGTTT***lat5-1* **AAGGTTTCCATCATCCCACTCATTTTCCTCATATTCTATGAAGTTTCTGGGGGTCCGTTT***lat5-2* **AAGGTTTCCATCATCCCACTCATTTTCCTCATATTCTATGAAGTTTCTGGGGGTCCGTTT** ....:..370....:..380....:..390....:..400....:..410....:..420
*LAT5*  **GGGATTGAGGATAGTGTCAAGGCTGCTGGCCCACTCCTAGCAATTGCTGGATTTCTGCTG***lat5-1* **GGGATTGAGGATAGTGTCAAGGCTGCTGGCCCACTCCTAGCA----------------TG***lat5-2* **GGGATTGAGGATAGTGTCAAGGCTGCTGGCCCACTCCTAGCAATTGCTGGATTTCTGCTG** ....:..430....:..440....:..450....:..460....:..470....:..480
*LAT5*  **TTTGCACTCATATGGAGTGTCCCGGAAGCCCTGATTACTGCAGAGATGGGCACTATGTTT***lat5-1* **ATCACAATT-TCTGGAGTGTCCCGGAAGCCCTGATTACTGCAGAGATGGGCACTATGTTT***lat5-2* **TTTGCACTCATATGGAGTGTCCCGGAAGCCCTGATTACTGCAGAGATGGGCACTATGTTT** ....:..490....:..500....:..510....:..520....:..530....:..540
*LAT5*  **CCTGAGAATGGTGGTTACGTCGTCTGGGTCTCTTCAGCCCTTGGGCCATTCTGGGGTTTT***lat5-1* **CCTGAGAATGGTGGTTACGTCGTCTGGGTCTCTTCAGCCCTTGGGCCATTCTGGGGTTTT***lat5-2* **CCTGAGAATGGTGGTTACGTCGTCTGGGTCTCTTCAGCCCTTGGGCCATTCTGGGGTTTT** ....:..550....:..560....:..570....:..580....:..590....:..600
*LAT5*  **CAGCAAGGCTGGGCAAAGTGGCTGAGTGGTGTCATAGATAATGCTCTCTATCCAGTCCTC***lat5-1* **CAGCAAGGCTGGGCAAAGTGGCTGAGTGGTGTCATAGATAATGCTCTCTATCCAGTCCTC***lat5-2* **CAGCAAGGCTGGGCAAAGTGGCTGAGTGGTGTCATAGATAATGCTCTCTATCCAGTCCTC** ....:..610....:..620....:..630....:..640....:..650....:..660
*LAT5*  **TTCCTCGACTATGTTAAGTCCAGCATTCCAGCTCTTGGAGGTGGTCTCCCAAGGACCTTG***lat5-1* **TTCCTCGACTATGTTAAGTCCAGCATTCCAGCTCTTGGAGGTGGTCTCCCAAGGACCTTG***lat5-2* **TTCCTCGACTATGTTAAGTCCAGCATTCCAGCTCTTGGAGGTGGTCTCCCAAGGACCTTG** ....:..670....:..680....:..690....:..700....:..710....:..720
*LAT5*  **GCGGTGCTTATCCTCACAGTTGCACTTACTTACATGAACTACAGAGGGTTGACAATAGTT***lat5-1* **GCGGTGCTTATCCTCACAGTTGCACTTACTTACATGAACTACAGAGGGTTGACAATAGTT***lat5-2* **GCGGTGCTTATCCTCACAGTTGCACTTACTTACATGAACTACAGAGGGTTGACAATAGTT**

....:..730....:..740....:..750....:..760....:..770....:..780
*LAT5*  **GGCTGGGTGGCAGTCTTTCTTGGCGTGTTCTCTCTACTCCCGTTTTTTGTTATGGGATTA***lat5-1* **GGCTGGGTGGCAGTCTTTCTTGGCGTGTTCTCTCTACTCCCGTTTTTTGTTATGGGATTA***lat5-2* **GGCTGGGTGGCAGTCTTTCTTGGCGTGTTCTCTCTACTCCCGTTTTTTGTTATGGGATTA** ....:..790....:..800....:..810....:..820....:..830....:..840
*LAT5*  **ATAGCTATTCCCCGAATCGAACCCTCAAGATGGCTTGAAATGGACTTGGGGAATGTGAAT***lat5-1* **ATAGCTATTCCCCGAATCGAACCCTCAAGATGGCTTGAAATGGACTTGGGGAATGTGAAT***lat5-2* **ATAGCTATTCCCCGAATCGAACCCTCAAGATGGCTTGAAATGGACTTGGGGAATGTGAAT** ....:..850....:..860....:..870....:..880....:..890....:..900
*LAT5*  **TGGGGTTTATATCTAAACACACTGTTTTGGAACCTCAATTATTGGGACTCAATCAGTACC***lat5-1* **TGGGGTTTATATCTAAACACACTGTTTTGGAACCTCAATTATTGGGACTCAATCAGTACC***lat5-2* **TGGGGTTTATATCTAAACACACTGTTTTGGAACCTCAATTATTGGGACTCAATCAGTACC** ....:..910....:..920....:..930....:..940....:..950....:..960
*LAT5*  **CTTGCTGGAGAGGTTGAGAATCCAAAGAGAACACTCCCAAGGGCACTTTCTTATGCTCTA***lat5-1* **CTTGCTGGAGAGGTTGAGAATCCAAAGAGAACACTCCCAAGGGCACTTTCTTATGCTCTA***lat5-2* **CTTGCTGGAGAGGTTGAGAATCCAAAGAGAACACTCCCAAGGGCACTTTCTTATGCTCTA** ....:..970....:..980....:..990....:.1000....:.1010....:.1020
*LAT5*  **GTTTTAGTGGTGGGGGGATACCTCTACCCTCTGATCACCTGTACAGCAGCAGTTCCAGTT***lat5-1* **GTTTTAGTGGTGGGGGGATACCTCTACCCTCTGATCACCTGTACAGCAGCAGTTCCAGTT***lat5-2* **GTTTTAGTGGTGGGGGGATACCTCTACCCTCTGATCACCTGTACAGCAGCAGTTCCAGTT** ....:.1030....:.1040....:.1050....:.1060....:.1070....:.1080
*LAT5*  **GTTCGGGAGTTCTGGACGGATGGATATTTCTCAGACGTTGCGAGAATTCTTGGTGGTTTC***lat5-1* **GTTCGGGAGTTCTGGACGGATGGATATTTCTCAGACGTTGCGAGAATTCTTGGTGGTTTC***lat5-2* **GTTCGGGAGTTCTGGACGGATGGATATTTCTCAGACGTTGCGAGAATTCTTGGTGGTTTC** ....:.1090....:.1100....:.1110....:.1120....:.1130....:.1140
*LAT5*  **TGGTTGCACTCGTGGCTTCAAGCAGCTGCTGCACTGTCCAACATGGGCAATTTCGTAACT***lat5-1* **TGGTTGCACTCGTGGCTTCAAGCAGCTGCTGCACTGTCCAACATGGGCAATTTCGTAACT***lat5-2* **TGGTTGCACTCGTGGCTTCAAGCAGCTGCTGCACTGTCCAACATGGGCAATTTCGTAACT** ....:.1150....:.1160....:.1170....:.1180....:.1190....:.1200
*LAT5*  **GAAATGAGCAGTGATTCTTACCAGCTTCTCGGGATGGCTGAGCGTGGAATGCTTCCAGAG***lat5-1* **GAAATGAGCAGTGATTCTTACCAGCTTCTCGGGATGGCTGAGCGTGGAATGCTTCCAGAG***lat5-2* **GAAATGAGCAGTGATTCTTACCAGCTTCTCGGGATGGCTGAGCGTGGAATGCTTCCAGAG** ....:.1210....:.1220....:.1230....:.1240....:.1250....:.1260
*LAT5*  **TTTTTCGCCAAGAGATCTCGCTATGGAACCCCACTTATTGGCATCATGTTCTCCGCGTTT***lat5-1* **TTTTTCGCCAAGAGATCTCGCTATGGAACCCCACTTATTGGCATCATGTTCTCCGCGTTT***lat5-2* **TTTTTCGCCAAGAGATCTCGCTATGGAACCCCACTTATTGGCATCATGTTCTCCGCGTTT** ....:.1270....:.1280....:.1290....:.1300....:.1310....:.1320
*LAT5*  **GGTGTGGTCCTGCTGTCCTGGATGAGCTTCCAGGAGATCATCGCTGCGGAGAACTACCTG***lat5-1* **GGTGTGGTCCTGCTGTCCTGGATGAGCTTCCAGGAGATCATCGCTGCGGAGAACTACCTG***lat5-2* **GGTGTGGTCCTGCTGTCCTGGATGAGCTTCCAGGAGATCATCGCTGCGGAGAACTACCTG** ....:.1330....:.1340....:.1350....:.1360....:.1370....:.1380
*LAT5*  **TACTGCTTCGGTATGATCCTGGAATTCATCGCCTTCATCAAGCTGAGGGTGGTCCACCCA***lat5-1* **TACTGCTTCGGTATGATCCTGGAATTCATCGCCTTCATCAAGCTGAGGGTGGTCCACCCA***lat5-2* **TACTGCTTCGGTATGATCCTGGAATTCATCGCCTTCATCAAGCTGAGGGTGGTCCACCCA** ....:.1390....:.1400....:.1410....:.1420....:.1430....:.1440
*LAT5*  **AACGCCTCCCGACCTTACAAGATCCCACTGGGCACCATCGGCGCTGTCCTGATGATCATC***lat5-1* **AACGCCTCCCGACCTTACAAGATCCCACTGGGCACCATCGGCGCTGTCCTGATGATCATC***lat5-2* **AACGCCTCCCGACCTTACAAGATCCCACTGGGCACCATCGGCGCTGTCCTGATGATCATC**

....:.1450....:.1460....:.1470....:.1480....:.1490....:.1500
*LAT5*  **CCACCTACCATTCTGATCGTCGTGGTGATGATGCTCGCGTCCTTCAAGGTGATGGTGGTC***lat5-1* **CCACCTACCATTCTGATCGTCGTGGTGATGATGCTCGCGTCCTTCAAGGTGATGGTGGTC***lat5-2* **CCACCTACCATTCTGATCGTCGTGGTGATGATGCTCGCGTCCTTCAAGGTGATGGTGGTC** ....:.1510....:.1520....:.1530....:.1540....:.1550....:.1560
*LAT5*  **AGCATCATGGCAATGCTGGTTGGGTTCGTGCTGCAGCCGGCTCTGGTGTACGTGGAGAAG***lat5-1* **AGCATCATGGCAATGCTGGTTGGGTTCGTGCTGCAGCCGGCTCTGGTGTACGTGGAGAAG***lat5-2* **AGCATCATGGCAATGCTGGTTGGGTTCGTGCTGCAGCCGGCTCTGGTGTACGTGGAGAAG** ....:.1570....:.1580....:.1590....:.1600....:.1610....:.1620
*LAT5*  **AGACGGTGGCTGAAGTTCTCCATAAGCGCAGAACTGCCAGATTTGCCGTACTCGAACGTT***lat5-1* **AGACGGTGGCTGAAGTTCTCCATAAGCGCAGAACTGCCAGATTTGCCGTACTCGAACGTT***lat5-2* **AGACGGTGGCTGAAGTTCTCCATAAGCGCAGAACTGCCAGATTTGCCGTACTCGAACGTT** ....:.1630....:.1640....:.1650...
*LAT5*  **GAGGAAGACAGCACAATCCCACTTGTGTGCTGA***lat5-1* **GAGGAAGACAGCACAATCCCACTTGTGTGCTGA***lat5-2* **GAGGAAGACAGCACAATCCCACTTGTGTGCTGA

*LAT7*/*PUT2* (Os12g0580400; LOC_Os12g39080)**

....:...10....:...20....:...30....:...40....:...50....:...60
*LAT7* **ATGACCGGAGCCTGCGAGGCGGCGCCGGCGCGGCGGCGGGGGCTGACGGTGCTCCCCCTC***lat7-1* **ATGACCGGAGCCTGCGAGGCGGCGCCGGCGCGGCGGCGGGGGCTGACGGTGCTCCCCCTC***lat7-2* **ATGACCGGAGCCTGCGAGGCGGCGCCGGCGCGGCGGCGGGGGCTGACGGTGCTCCCCCTC** ....:...70....:...80....:...90....:..100....:..110....:..120
*LAT7*  **GTCGCGCTCATCTTCTACGACGTGTCGGGGGGCCCCTTCGGCATCGAGGACTCGGTCCGC***lat7-1* **GTCGCGCTCATCTTCTACGACGTGTCGGGGGGCCCCTTCGGCATCGAGGACTCGGTCCGC***lat7-2* **GTCGCGCTCATCTTCTACGACGTGTCGGGGGGCCCCTTCGGCATCGAGGACTCGGTCCGC** ....:..130....:..140....:..150....:..160....:..170....:..180
*LAT7*  **GCCGGCGGCGGCGCGCTCCTCCCGATCCTGGGGTTCCTCGTCCTCCCCGTGCTCTGGTCC***lat7-1* **GCCGGCGGCGGCGCGCTCCTCCCGATCCTGGGGTTCCTCGTCCTCCCCGTGCTCTGGTCC***lat7-2* **GCCGGCGGCGGCGCGCTCCTCCCGATCCTGGGGTTCCTCGTCCTCCCCGTGCTCTGGTC-** ....:..190....:..200....:..210....:..220....:..230....:..240
*LAT7*  **CTCCCCGAGGCGCTCGTCACCGCCGAGCTCGCCTCCGCGTTCCCCACCAACGCCGGCTAC***lat7-1* **CT-CCCGAGGCGCTCGTCACCGCCGAGCTCGCCTCCGCGTTCCCCACCAACGCCGGCTAC***lat7-2* **---CCCGAGGCGCTCGTCACCGCCGAGCTCGCCTCCGCGTTCCCCACCAACGCCGGCTAC** ....:..250....:..260....:..270....:..280....:..290....:..300
*LAT7*  **GTCGCCTGGGTCTCCGCCGCGTTCGGCCCCGCCGCGGCGTTCCTCGTCGGGTTCTCCAAG***lat7-1* **GTCGCCTGGGTCTCCGCCGCGTTCGGCCCCGCCGCGGCGTTCCTCGTCGGGTTCTCCAAG***lat7-2* **GTCGCCTGGGTCTCCGCCGCGTTCGGCCCCGCCGCGGCGTTCCTCGTCGGGTTCTCCAAG** ....:..310....:..320....:..330....:..340....:..350....:..360
*LAT7*  **TGGGCGTCGGGGACGCTCGACAACGCGCTCTACCCGGTGCTCTTCCTCGACTACCTCCGC***lat7-1* **TGGGCGTCGGGGACGCTCGACAACGCGCTCTACCCGGTGCTCTTCCTCGACTACCTCCGC***lat7-2* **TGGGCGTCGGGGACGCTCGACAACGCGCTCTACCCGGTGCTCTTCCTCGACTACCTCCGC** ....:..370....:..380....:..390....:..400....:..410....:..420
*LAT7*  **TCCGGCGGGGGGCTCGTGCTCTCCCCGCCGGCCCGCTCCCTCGCCGTGCTCGCGCTCACC***lat7-1* **TCCGGCGGGGGGCTCGTGCTCTCCCCGCCGGCCCGCTCCCTCGCCGTGCTCGCGCTCACC***lat7-2* **TCCGGCGGGGGGCTCGTGCTCTCCCCGCCGGCCCGCTCCCTCGCCGTGCTCGCGCTCACC** ....:..430....:..440....:..450....:..460....:..470....:..480
*LAT7*  **GCCGCGCTCACCTACCTCAACTTCCGGGGGCTCCACCTCGTCGGCCTCTCCGCGCTGGCG***lat7-1* **GCCGCGCTCACCTACCTCAACTTCCGGGGGCTCCACCTCGTCGGCCTCTCCGCGCTGGCG***lat7-2* **GCCGCGCTCACCTACCTCAACTTCCGGGGGCTCCACCTCGTCGGCCTCTCCGCGCTGGCG** ....:..490....:..500....:..510....:..520....:..530....:..540
*LAT7*  **CTCACCGCGTTCTCGCTCTCCCCGTTCGTCGCGCTCGCCGTGCTCGCCGCCCCCAAGATC***lat7-1* **CTCACCGCGTTCTCGCTCTCCCCGTTCGTCGCGCTCGCCGTGCTCGCCGCCCCCAAGATC***lat7-2* **CTCACCGCGTTCTCGCTCTCCCCGTTCGTCGCGCTCGCCGTGCTCGCCGCCCCCAAGATC** ....:..550....:..560....:..570....:..580....:..590....:..600
*LAT7*  **CGCCCGTCGCGGTGGCTCGCCGTGAACGTGGCCGCCGTTGAGCCGCGCGCCTACTTCAAC***lat7-1* **CGCCCGTCGCGGTGGCTCGCCGTGAACGTGGCCGCCGTTGAGCCGCGCGCCTACTTCAAC***lat7-2* **CGCCCGTCGCGGTGGCTCGCCGTGAACGTGGCCGCCGTTGAGCCGCGCGCCTACTTCAAC** ....:..610....:..620....:..630....:..640....:..650....:..660
*LAT7*  **TCCATGTTCTGGAACCTCAACTACTGGGACAAGGCGAGCACGCTTGCCGGCGAGGTGGAG***lat7-1* **TCCATGTTCTGGAACCTCAACTACTGGGACAAGGCGAGCACGCTTGCCGGCGAGGTGGAG***lat7-2* **TCCATGTTCTGGAACCTCAACTACTGGGACAAGGCGAGCACGCTTGCCGGCGAGGTGGAG** ....:..670....:..680....:..690....:..700....:..710....:..720
*LAT7*  **GAGCCGAGGAAGACGTTCCCGAAGGCGGTGTTCGGCGCGGTGGGGCTCGTCGTGGGCGCG***lat7-1* **GAGCCGAGGAAGACGTTCCCGAAGGCGGTGTTCGGCGCGGTGGGGCTCGTCGTGGGCGCG***lat7-2* **GAGCCGAGGAAGACGTTCCCGAAGGCGGTGTTCGGCGCGGTGGGGCTCGTCGTGGGCGCG**

....:..730....:..740....:..750....:..760....:..770....:..780
*LAT7*  **TACCTCATCCCGCTCCTCGCCGGGACGGGCGCGCTGCCGTCGGAGACGGCGGGGGAGTGG***lat7-1* **TACCTCATCCCGCTCCTCGCCGGGACGGGCGCGCTGCCGTCGGAGACGGCGGGGGAGTGG***lat7-2* **TACCTCATCCCGCTCCTCGCCGGGACGGGCGCGCTGCCGTCGGAGACGGCGGGGGAGTGG** ....:..790....:..800....:..810....:..820....:..830....:..840
*LAT7*  **ACGGACGGGTTCTTCTCCGTGGTCGGCGACCGGATCGGCGGGCCGTGGCTGCGCGTGTGG***lat7-1* **ACGGACGGGTTCTTCTCCGTGGTCGGCGACCGGATCGGCGGGCCGTGGCTGCGCGTGTGG***lat7-2* **ACGGACGGGTTCTTCTCCGTGGTCGGCGACCGGATCGGCGGGCCGTGGCTGCGCGTGTGG** ....:..850....:..860....:..870....:..880....:..890....:..900
*LAT7*  **ATCCAGGCCGCCGCGGCCATGTCCAACATGGGGCTCTTCGAGGCCGAGATGAGCGGCGAC***lat7-1* **ATCCAGGCCGCCGCGGCCATGTCCAACATGGGGCTCTTCGAGGCCGAGATGAGCGGCGAC***lat7-2* **ATCCAGGCCGCCGCGGCCATGTCCAACATGGGGCTCTTCGAGGCCGAGATGAGCGGCGAC** ....:..910....:..920....:..930....:..940....:..950....:..960
*LAT7*  **TCGTTCCAGCTCCTCGGCATGGCGGAGATGGGCATGATCCCGGCGATCTTCGCGCGCAGG***lat7-1* **TCGTTCCAGCTCCTCGGCATGGCGGAGATGGGCATGATCCCGGCGATCTTCGCGCGCAGG***lat7-2* **TCGTTCCAGCTCCTCGGCATGGCGGAGATGGGCATGATCCCGGCGATCTTCGCGCGCAGG** ....:..970....:..980....:..990....:.1000....:.1010....:.1020
*LAT7*  **TCGCGCCACGGCACGCCGACGTACAGCATCCTCTGCTCGGCCACCGGCGTCGTCATCCTC***lat7-1* **TCGCGCCACGGCACGCCGACGTACAGCATCCTCTGCTCGGCCACCGGCGTCGTCATCCTC***lat7-2* **TCGCGCCACGGCACGCCGACGTACAGCATCCTCTGCTCGGCCACCGGCGTCGTCATCCTC** ....:.1030....:.1040....:.1050....:.1060....:.1070....:.1080
*LAT7*  **TCCTTCATGAGCTTCCAGGAGATCGTCGAGTTCCTCAACTTCCTCTACGGCCTCGGGATG***lat7-1* **TCCTTCATGAGCTTCCAGGAGATCGTCGAGTTCCTCAACTTCCTCTACGGCCTCGGGATG***lat7-2* **TCCTTCATGAGCTTCCAGGAGATCGTCGAGTTCCTCAACTTCCTCTACGGCCTCGGGATG** ....:.1090....:.1100....:.1110....:.1120....:.1130....:.1140
*LAT7*  **CTCGCCGTGTTCGCCGCCTTCGTCAAGCTCCGCGTCAAGGACCCCGACCTCCCCCGCCCG***lat7-1* **CTCGCCGTGTTCGCCGCCTTCGTCAAGCTCCGCGTCAAGGACCCCGACCTCCCCCGCCCG***lat7-2* **CTCGCCGTGTTCGCCGCCTTCGTCAAGCTCCGCGTCAAGGACCCCGACCTCCCCCGCCCG** ....:.1150....:.1160....:.1170....:.1180....:.1190....:.1200
*LAT7*  **TACCGGATCCCCGTCGGCGCCGCGGGCGCCGCCGCCATGTGCGTCCCGCCCGTCGTCCTC***lat7-1* **TACCGGATCCCCGTCGGCGCCGCGGGCGCCGCCGCCATGTGCGTCCCGCCCGTCGTCCTC***lat7-2* **TACCGGATCCCCGTCGGCGCCGCGGGCGCCGCCGCCATGTGCGTCCCGCCCGTCGTCCTC** ....:.1210....:.1220....:.1230....:.1240....:.1250....:.1260
*LAT7*  **ATCACCACCGTCATGTGCCTCGCCTCCGCCAGGACGCTCGTCGTCAGCGCCGCCGTGGCC***lat7-1* **ATCACCACCGTCATGTGCCTCGCCTCCGCCAGGACGCTCGTCGTCAGCGCCGCCGTGGCC***lat7-2* **ATCACCACCGTCATGTGCCTCGCCTCCGCCAGGACGCTCGTCGTCAGCGCCGCCGTGGCC** ....:.1270....:.1280....:.1290....:.1300....:.1310....:.1320
*LAT7*  **GTCGCCGGCGTCGCCATGTACTACGGCGTCGAGCACATGAAGGCCACCGGCTGCGTCGAG***lat7-1* **GTCGCCGGCGTCGCCATGTACTACGGCGTCGAGCACATGAAGGCCACCGGCTGCGTCGAG***lat7-2* **GTCGCCGGCGTCGCCATGTACTACGGCGTCGAGCACATGAAGGCCACCGGCTGCGTCGAG** ....:.1330....:.1340....:.1350....:.1360....:.1370....:.1380
*LAT7*  **TTCTTGACGCCGGTGCCGCCTGACAGCCTCCGTGGATCATCATCATCATCATCCTCATCG***lat7-1* **TTCTTGACGCCGGTGCCGCCTGACAGCCTCCGTGGATCATCATCATCATCATCCTCATCG***lat7-2* **TTCTTGACGCCGGTGCCGCCTGACAGCCTCCGTGGATCATCATCATCATCATCCTCATCG** ....:.1390....:.1400....:.1410....:.1420....:.1430....:.1440
*LAT7*  **GCAGCGTCCGACAACGGCGGCGACGACGACGTCGAGGACGTCTGCGCCCTCCTCCTCGCC***lat7-1* **GCAGCGTCCGACAACGGCGGCGACGACGACGTCGAGGACGTCTGCGCCCTCCTCCTCGCC***lat7-2* **GCAGCGTCCGACAACGGCGGCGACGACGACGTCGAGGACGTCTGCGCCCTCCTCCTCGCC**

....:.1450....:.1460....:.1470....:.1480....:.1490.
*LAT7*  **GCCGGCGAGCACGCCGGAGAAGGCGTCAGTGTCAGCAAGGAGAATTATTAG***lat7-1* **GCCGGCGAGCACGCCGGAGAAGGCGTCAGTGTCAGCAAGGAGAATTATTAG***lat7-2* **GCCGGCGAGCACGCCGGAGAAGGCGTCAGTGTCAGCAAGGAGAATTATTAG**
