# Supplementary material for: Methylviologen resistance in loss-of-function mutants of the polyamine transporter gene OsLAT5
Source: PLoS One. 2026 Apr 16;21(4):e0346828. doi: 10.1371/journal.pone.0346828 (PMC13086316; doi:10.1371/journal.pone.0346828)
Supplement: S6 File — (DOCX) [file pone.0346828.s006.docx]

**Supporting Information S6. Putative rice Lat1, Lat5, and Lat7 protein sequences from single mutant lines.**

Putative lat1-1 protein sequence:

MADTGGRPEVSLATVRSPGHPAASTTAAAAADLGHADTGQEKPTVESAQPANGAAPMGECGTEYRGLPDGDAGGPMPSSARTVSMIPLIFLIFYEVSGGPFGIEDSVGAAGPLLAIIGFLVLPVIWSIPEALITAELGAMFPENGGYVVWVASALGPYWGFQQGWMKWLSGVIDNALYPVLFLDYLKSGVPALGGGAPRAFAVVGLTAVLTLLNYRGLTVVGWVAICLGVFSLLPFFVMGLIALPKLRPARWLVIDLHNVDWNLYLNTLFWNLNYWDSISTLAGEVKNPGKTLPKALFYAVIFVVVAYLYPLLAGTGAVPLDRGQWTDGYFADIAKLLGGAWLMWWVQSAAALSNMGMFVAEMSSDSYAGHGGAGHAPVLLRGAVAVRHAAGGHPLLGLRRAAALDDELPGDRGGRELPLLLRHAPRVRRLHPAPGEAPRRGAPIQGAAGHSRVRGDAGAADGADRRGARAVHAEGGGGEPRRGGHGARAAAGAEVRGEEAVAEVLR*

Putative lat1-2 protein sequence:

MADTGGRPEVSLATVRSPGHPAASTTAAAAADLGHADTGQEKPTVESAQPANGAAPMGECGTEYRGLPDGDAGGPMPSSARTVSMIPLIFLIFYEVSGGPFGIEDSVGAAGPLLAIIGFLVLPVIWSIPEALITAELGAMFPENGGYVVWVASALGPYWGFQQGWMKWLSGVIDNALYPVLFLDYLKSGVPALGGGAPRAFAVVGLTAVLTLLNYRGLTVVGWVAICLGVFSLLPFFVMGLIALPKLRPARWLVIDLHNVDWNLYLNTLFWNLNYWDSISTLAGEVKNPGKTLPKALFYAVIFVVVAYLYPLLAGTGAVPLDRGQWTDGYFADIAKLLGGAWLMWWVQSAAALSNMGMFVAEMSSDSYQLAGHGGAGHAPVLLRGAVAVRHAAGGHPLLGLRRAAALDDELPGDRGGRELPLLLRHAPRVRRLHPAPGEAPRRGAPIQGAAGHSRVRGDAGAADGADRRGARAVHAEGGGGEPRRGGHGARAAAGAEVRGEEAVAEVLR*

Putative lat5-1 protein sequence: MTNAWISPSVVALCPSPLPSSRLPGSVLSCWPDSRGIRRGAGEGTAGQTLRPARGFTVEKLRNTAITRANSACLPMEDCVGIKYSSVNEGEERKGAMASQRFPSSHSFSSYSMKFLGVRLGLRIVSRLLAHS*

Putative lat5-2 protein sequence:

MTNAWISPSVVALCPSPLPSSRLPGSVLSCWPDSRGIRRGAGEGTAGQTLRPARGFTVEKLRNTAITRANSACLPMEDCVGIKYSSVNEGEERKGASQRFPSSHSFSSYSMKFLGVRLGLRIVSRLLAHS*

Putative lat7-1 protein sequence:

MTGACEAAPARRRGLTVLPLVALIFYDVSGGPFGIEDSVRAGGGALLPILGFLVLPVLWSLPRRSSPPSSPPRSPPTPATSPGSPPRSAPPRRSSSGSPSGRRGRSTTRSTRCSSSTTSAPAGGSCSPRRPAPSPCSRSPPRSPTSTSGGSTSSASPRWRSPRSRSPRSSRSPCSPPPRSARRGGSP*

Putative lat7-2 protein sequence:

MTGACEAAPARRRGLTVLPLVALIFYDVSGGPFGIEDSVRAGGGALLPILGFLVLPVLWSPRRSSPPSSPPRSPPTPATSPGSPPRSAPPRRSSSGSPSGRRGRSTTRSTRCSSSTTSAPAGGSCSPRRPAPSPCSRSPPRSPTSTSGGSTSSASPRWRSPRSRSPRSSRSPCSPPPRSARRGGSP*
